# Data on peptidyl platform-based anticancer drug synthesis and triton-x-based micellar clusters (MCs) self-assembly peculiarities for enhanced solubilization, encapsulation of hydrophobic compounds and their interaction with HeLa cells

**DOI:** 10.1016/j.dib.2019.104052

**Published:** 2019-05-24

**Authors:** Alexey V. Solomonov, Yuriy S. Marfin, Evgeniy V. Rumyantsev, Elena Ragozin, Talia Shekhter Zahavi, Gary Gellerman, Alexander B. Tesler, Falk Muench, Akiko Kumagai, Atsushi Miyawaki

**Affiliations:** a Inorganic Chemistry Department, Ivanovo State University of Chemistry and Technology, 7 Sheremetevskij prosp., 153000, Ivanovo, Russian; b Ivanovo State Polytechnical University, 21 Sheremetevskij prosp., 153000, Ivanovo, Russian; c Department of Chemical Sciences, Ariel University of Samaria, Ariel, Israel; d Department of Molecular Microbiology and Biotechnology, George S. Wise Faculty of Life Sciences, Tel-Aviv University, Tel-Aviv 69978, Israel; e Department of Materials and Interfaces, Faculty of Chemistry, Weizmann Institute of Science, 76100, 234 Herzl Street, Rehovot, Israel; f Department of Materials and Earth Sciences, Technische Universität Darmstadt, Alarich-Weiss-Strasse 2, 64287, Darmstadt, Germany; g Centre of Brain Science, Laboratory for Cell Function and Dynamics, RIKEN, 2-1 Hirosawa, Wako, Saitama, 351-0198, Japan

**Keywords:** Micellar clusters, Surfactants, Triton-X, Self-assembly, Encapsulation, Anticancer drug synthesis, Fluorescence enhancement

## Abstract

The data presented here refer to a research article entitled “Self-Assembled Micellar Clusters Based on Triton-X-family Surfactants for Enhanced Solubilization, Encapsulation, Proteins Permeability Control, and Anticancer Drug Delivery” Solomonov et al., 2019. The present article provides the General Procedure for clusterization of Triton-X-based micelles and the effect of (i) metal ion, surfactant, and chelator concentration on the developed clusters formation, (ii) surfactant-chelator relation change, (iii) metal ion-micelles concertation ratio variation, (iv) metal ion replacement, (v) solvent replacement, (vi) kinetics of clusters formation, (vii) hydrophobic fluorescent dye (Coumarin 6) solubilization in aqueous MCs media, (viii) novel anticancer peptidyl drug synthesis and characterization and (ix) the viability of HeLa cells with and without the presence of drug-free Triton-X-based family MCs. These data provide additional insights useful for understanding all aspects of the micellar clusters formation, optimization, and control.

**Table:** Specifications table

Subject area	*Chemistry*
More specific subject area	*Chemistry of materials, surfactants, self-assembly*
Type of data	*Tables, figures*
How data was acquired	*Optical Microscopy: Micromed LUM-3 with digital camera ToupCam 5.0 MP CCD, Olympus BX-61* *Fluorescent spectroscopy: Cary Eclipse (Varian-Agilent, detector voltage is 600 V) and Horiba Jobin Yvon Fluorolog 3* *HPLC: ECOM semi-preparative system with TOPAZ dual UV detection* *Mass spectrometer: Agilent Infinity 1260*
Data format	*Raw, filtered, analyzed*
Experimental factors	*Optical Microscopy: Images were obtained with bright field mode* *Fluorescent spectroscopy: Spectra of MCs were taken in* 1 mm *cuvette*
Experimental features	*Fresh suspensions of all MCs were prepared before all experiments, especially with the cells* *The variations in the General Procedure in the MCs preparation have been used* *The fresh suspension of the MCs was directly added to the HeLa cells culture and incubated* *Novel anticancer drug has been synthesized*
Data source location	*Ivanovo State University of Chemistry and Technology, Russian Federation*
Data accessibility	*All data are available within this article*
Related research article	*Solomonov AV, Marfin YuS, Rumyantsev EV, Ragozin E, Shekhter-Zahavi T, Gellerman G, Tesler AB, Muench F, Kumagai A, Miyawaki A. Self-Assembled Micellar Clusters Based on Triton-X-family Surfactants for Enhanced Solubilization, Encapsulation, Proteins Permeability Control, and Anticancer Drug Delivery. Mater. Sci. Eng. C, 2019 99:794–804**[1]* https://www.sciencedirect.com/science/article/pii/S0928493118307379

**Table:** 

**Value of the data** • The data provide new insights into the micellar clusters synthesis based on Triton X-100 and X-114 surfactants, which can be of great value for researchers studying emulsions, surfactants, and self-assembly • The data describes several routes to micellar clusters self-assembly with a different optical and chemical composition by varying the preparation conditions that will be helpful for optimizing experimental conditions for future effective hydrophobic compounds encapsulation • The data describes the synthesis and characterization of newly developed anti-cancer PTR-58-CLB-CAMP peptide drug and demonstrates the viability of HeLa cells with and without the presence of drug-free Triton-X based micellar clusters, that may be relevant for future understanding differences in the interaction of other cell lines with the clusters • This data could be relevant for researchers specializing in the fields of interfacial chemistry and self-assembly and may be used in future for the development of novel anticancer drugs, drug carriers for targeted drug delivery or enhanced solubilization of hydrophobic compounds

## 1 Data

**Fig. 1:**
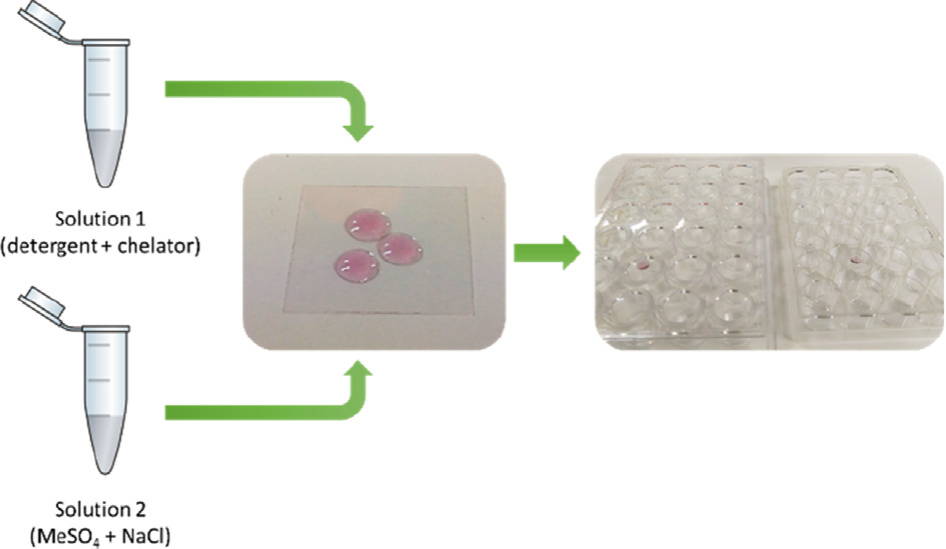
Preparation of the droplets by the General Procedure.

**Fig. 2:**
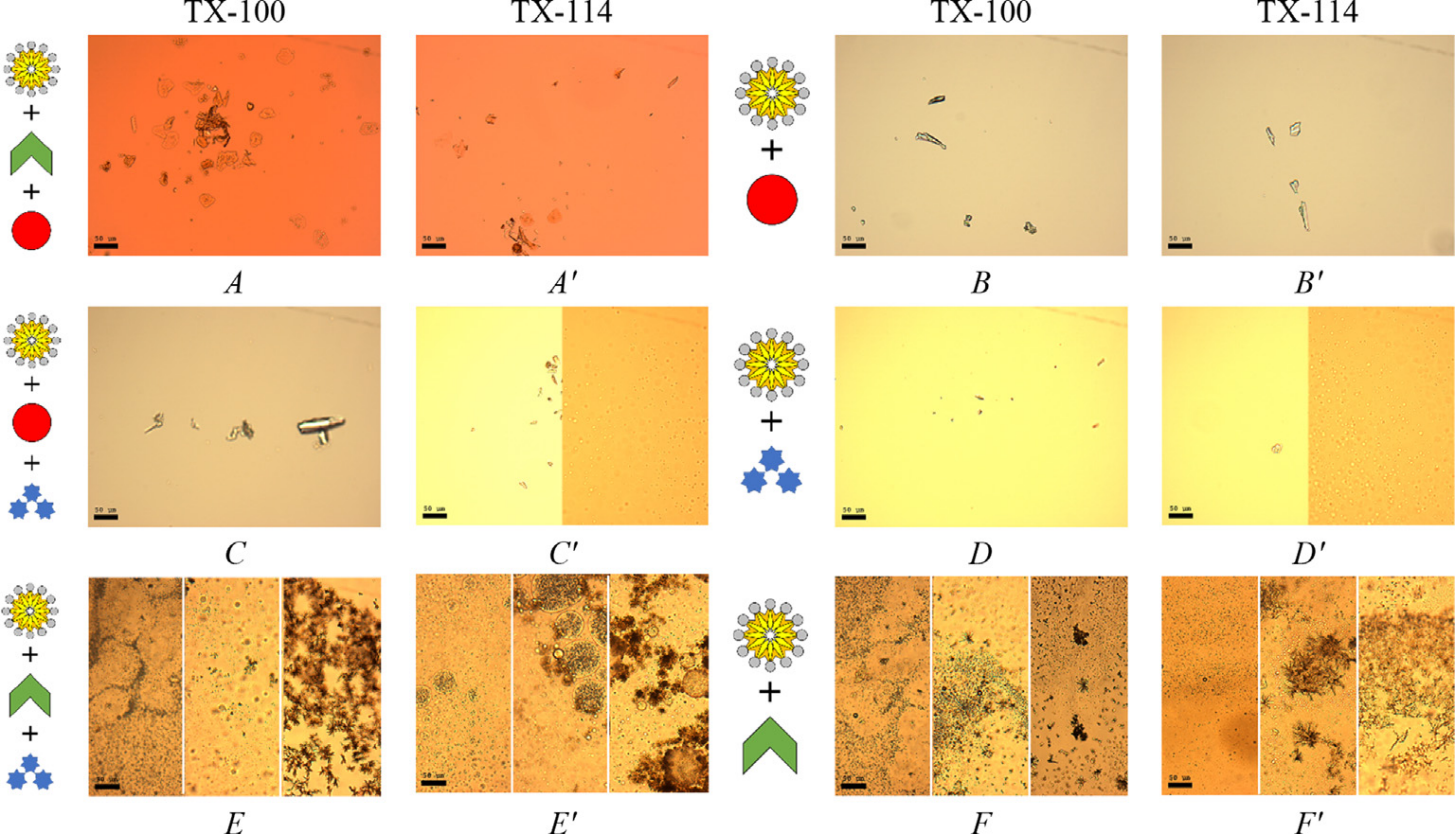
Effect of one or two components removing in the micellar solution. The effect of NaCl (*A*, *A′*), NaCl and BPhen (*B*, *B′*); BPhen (*C*, *C′*), BPhen and FeSO_4_ (*D*, *D′*); FeSO_4_ (*E*, *E′*), FeSO_4_ and NaCl (*F*, *F′*) removing. Scale bar is 50 μm.

**Fig. 3:**
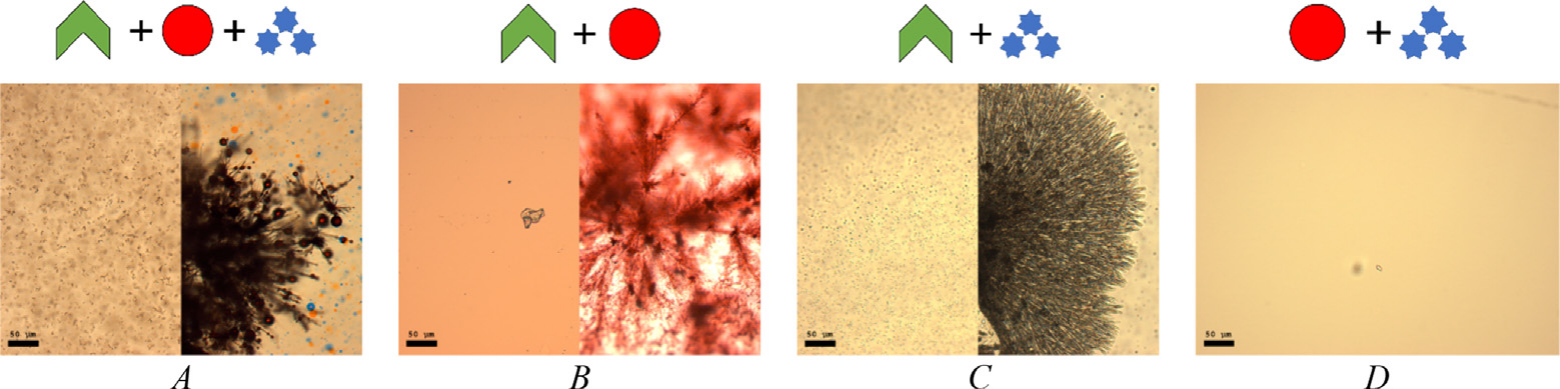
System response in the absence of surfactant micelles (*A*), micelles and NaCl (*B*), micelles and FeSO_4_ (*C*), micelles and BPhen (*D*). Scale bar is 50 μm.

**Fig. 4:**
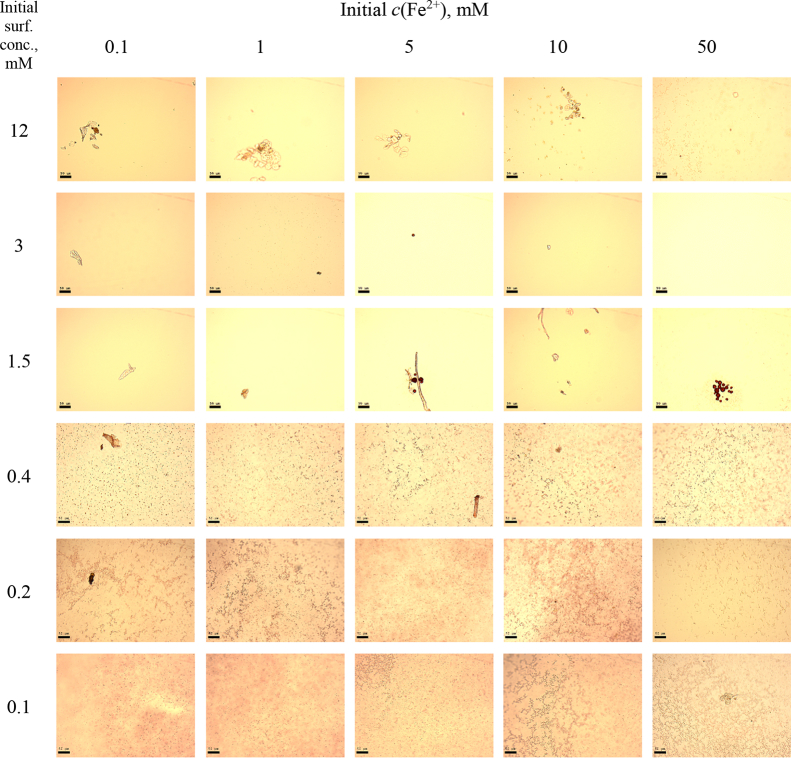
MCs based on TX-100 with initial concentrations of *c*(BPhen) = 5 mM and *c*(NaCl) = 200 mM, varying initial concentrations of surfactant and Fe^2+^ salt. Scale bar is 50 μm.

**Fig. 5:**
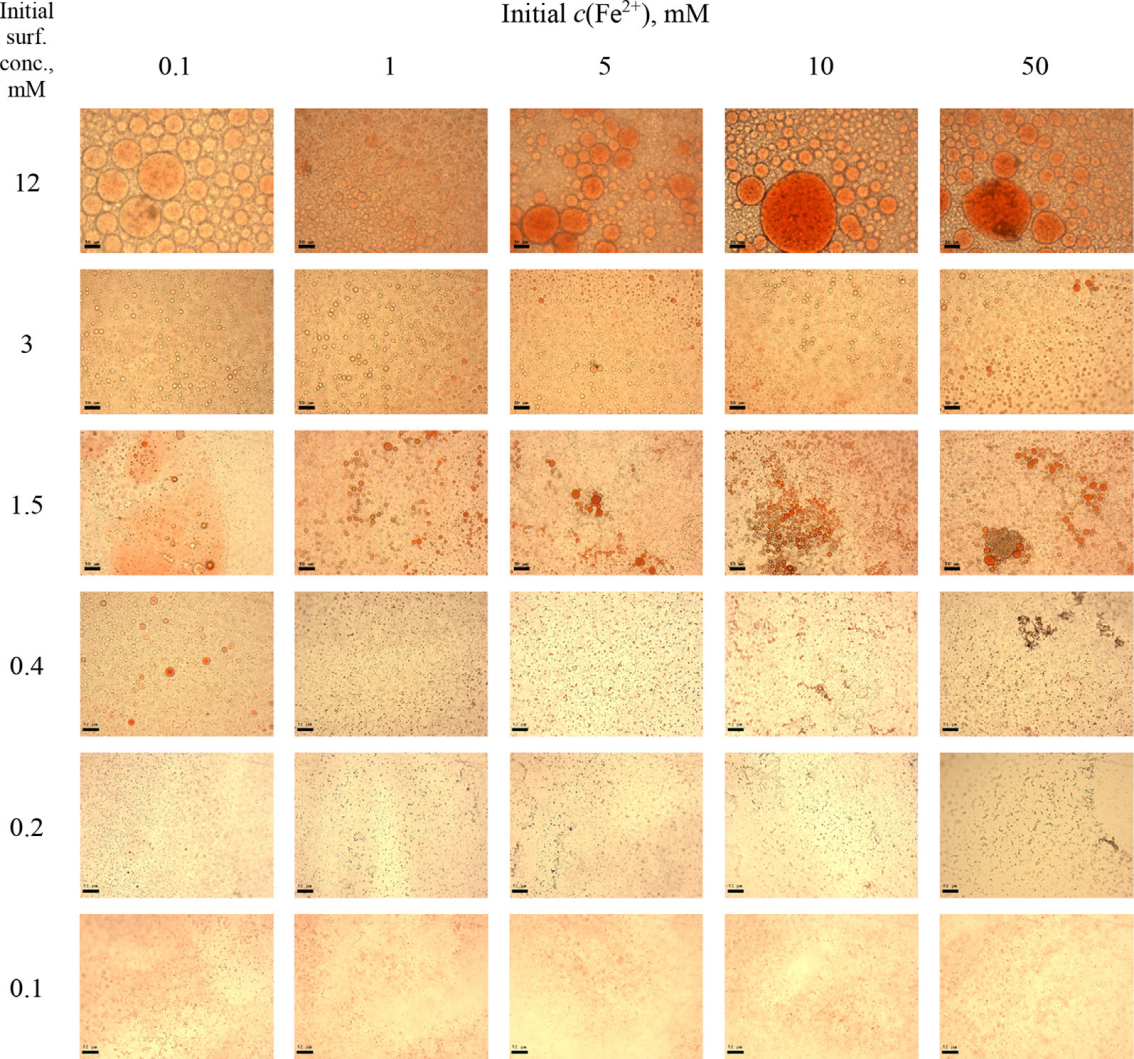
MCs based on TX-114 with initial concentrations of *c*(BPhen) = 5 mM and *c*(NaCl) = 200 mM, varying initial concentrations of surfactant and Fe^2+^ salt. Scale bar is 50 μm.

**Fig. 6:**
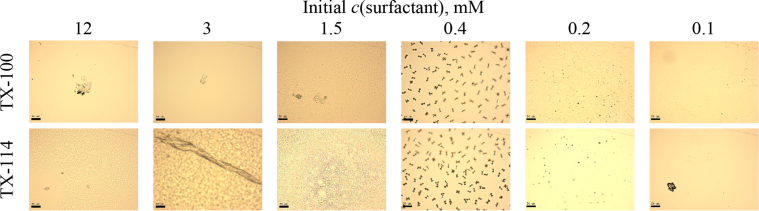
Control experiments without Fe^2+^ salt. Initial concentrations of *c*(BPhen) = 5 mM and *c*(NaCl) = 200 mM. Scale bar is 50 μm.

**Fig. 7:**
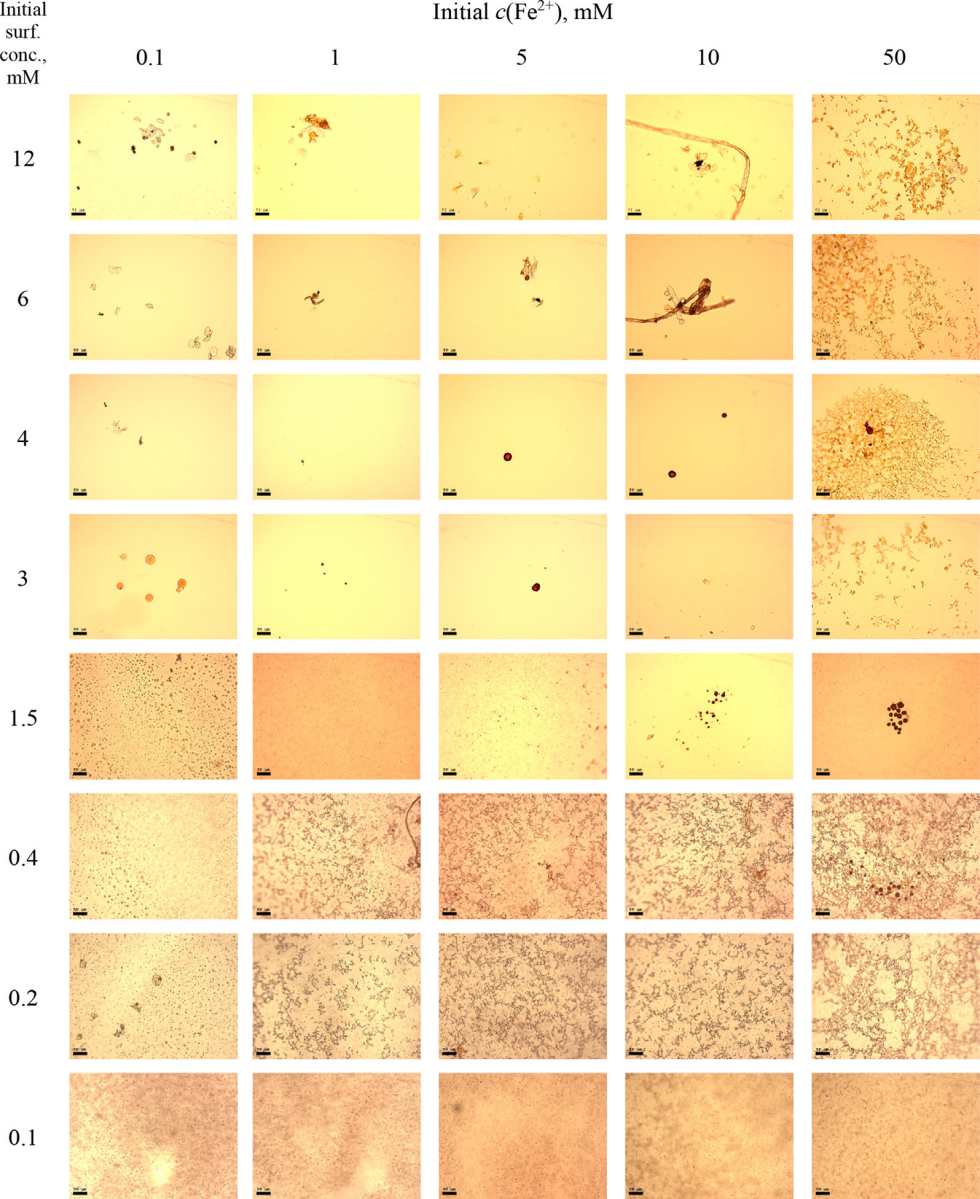
MCs based on TX-100 with initial concentrations of *c*(BPhen) = 10 mM and *c*(NaCl) = 200 mM, varying initial concentrations of the Triton and Fe^2+^ salt. Scale bar is 50 μm.

**Fig. 8:**
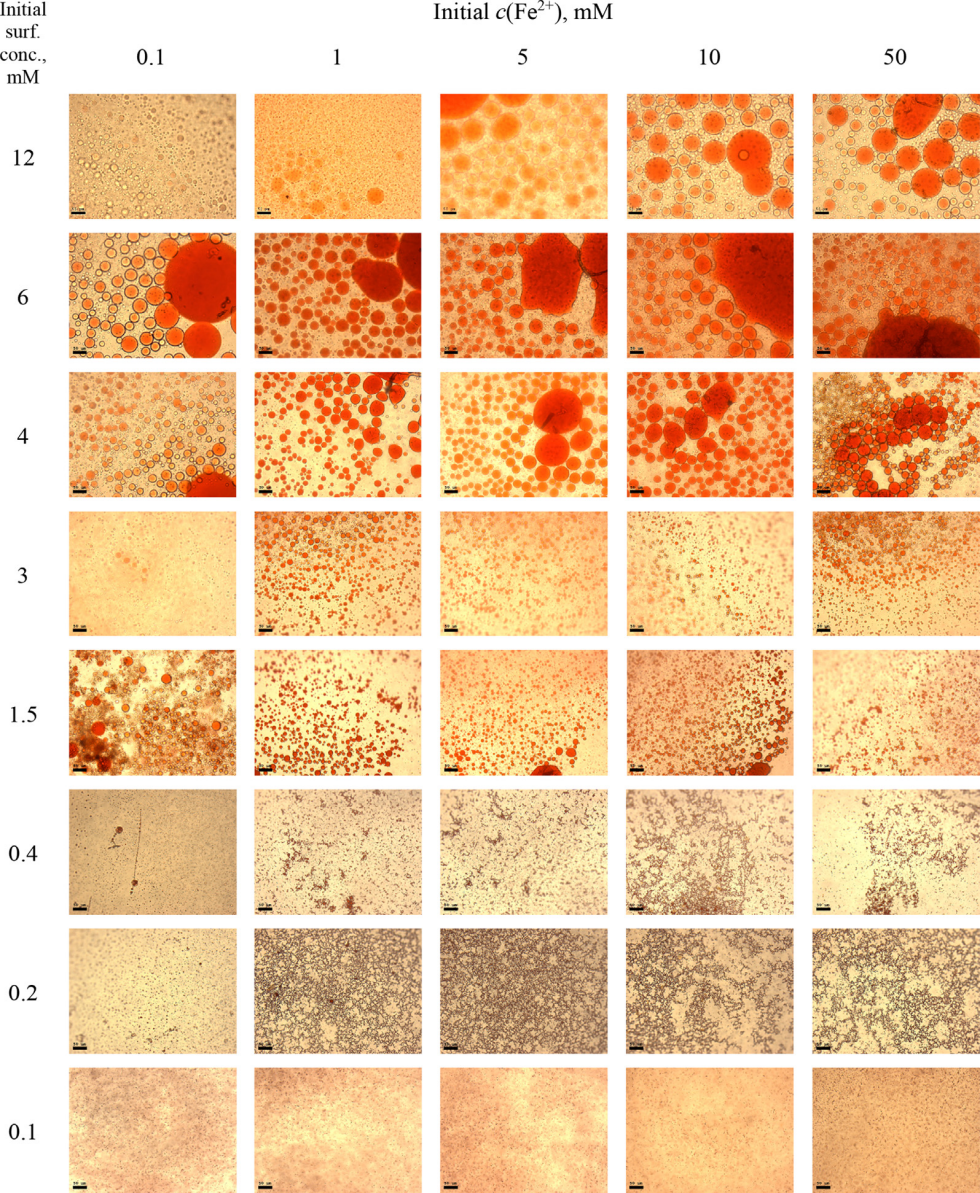
MCs based on TX-114 with initial concentrations of *c*(BPhen) = 10 mM and *c*(NaCl) = 200 mM, varying initial concentrations of the Triton and Fe^2+^ salt. Scale bar is 50 μm.

**Fig. 9:**
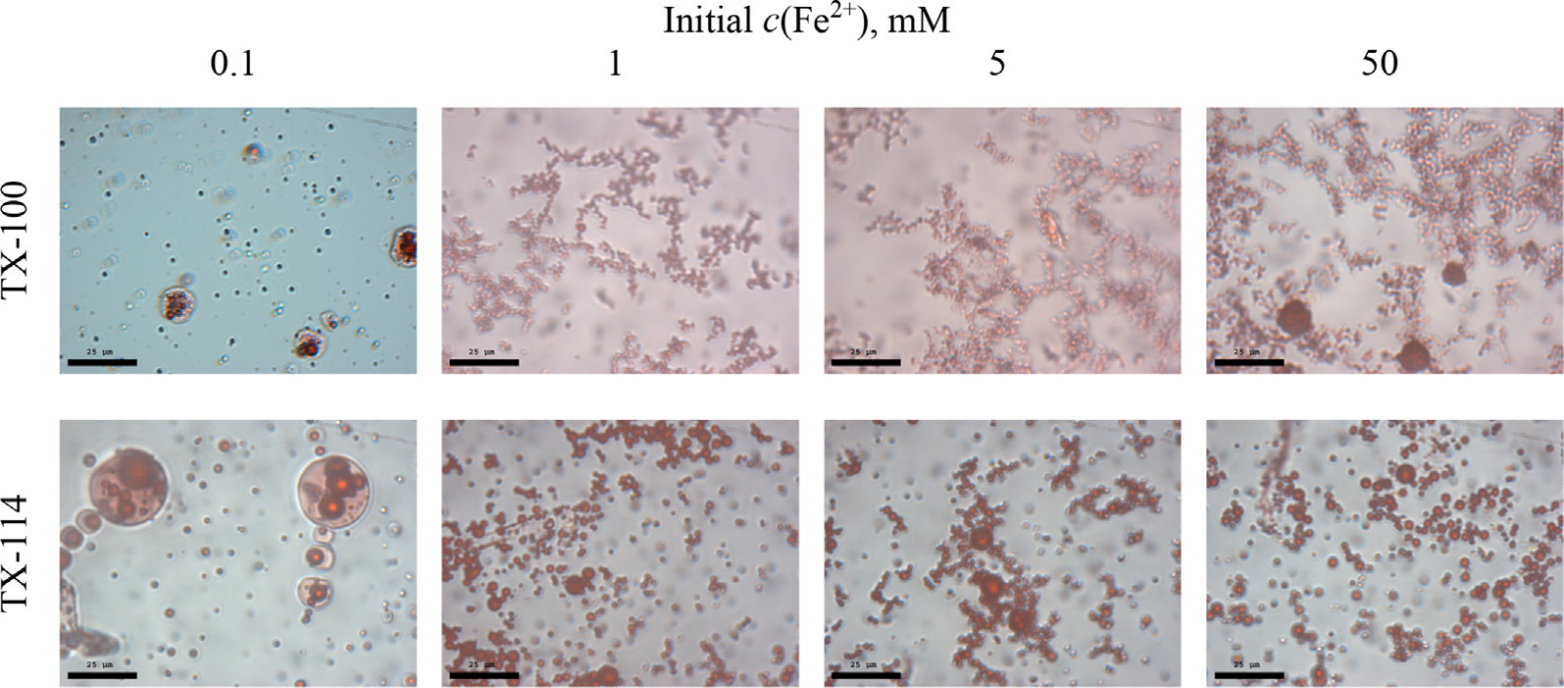
MCs based on TX-100/TX-114 with initial concentrations of surfactants of 0.4 mM, c(BPhen) = 10 mM and c(NaCl) = 200 mM; varying initial concentrations of surfactant and Fe^2+^ salt (increased magnification). Scale bar is 25 μm.

**Fig. 10:**
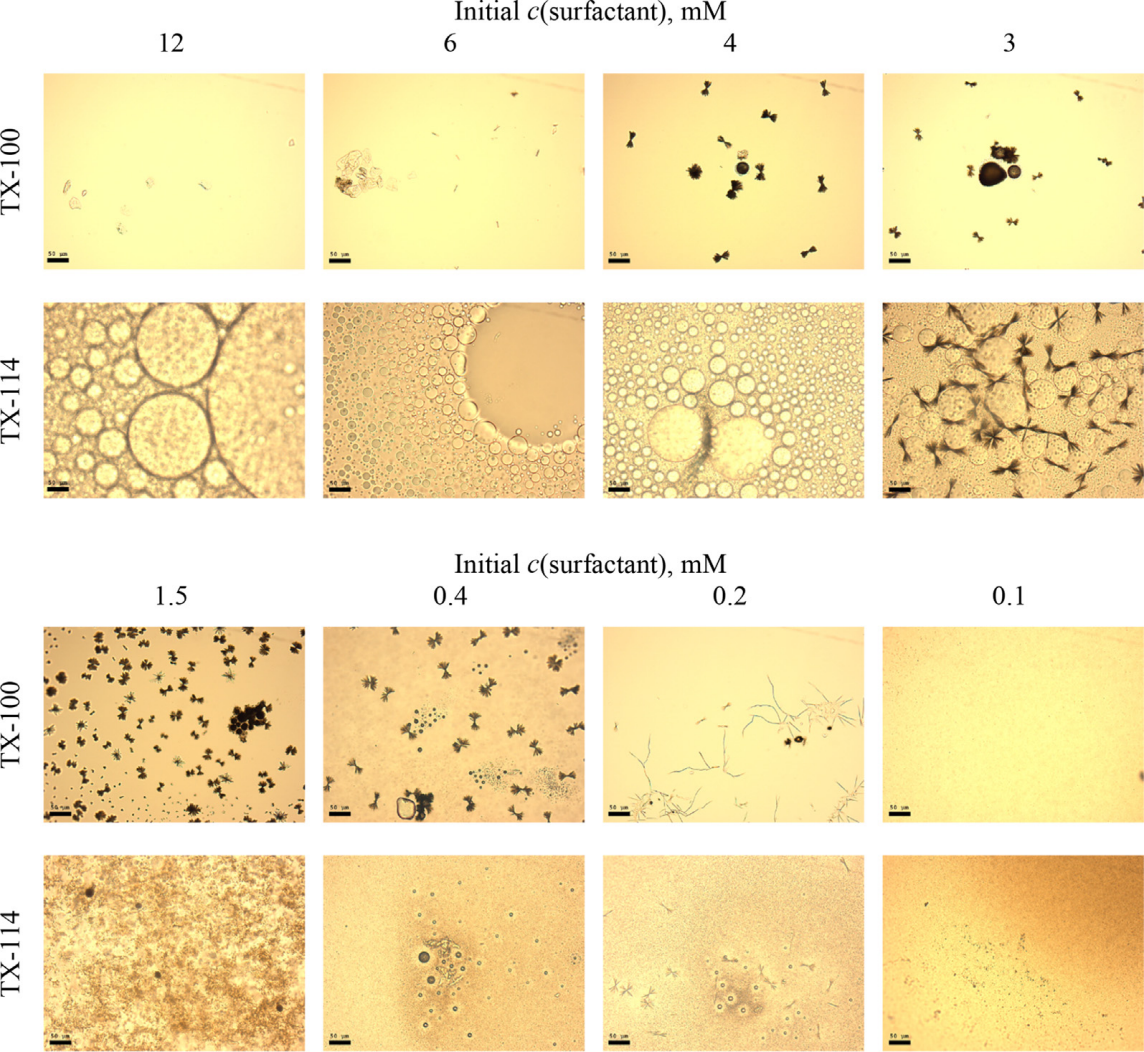
Control experiments without Fe^2+^ salt. Initial concentrations of *c*(BPhen) = 10 mM and *c*(NaCl) = 200 mM. Scale bar is 50 μm.

**Fig. 11:**
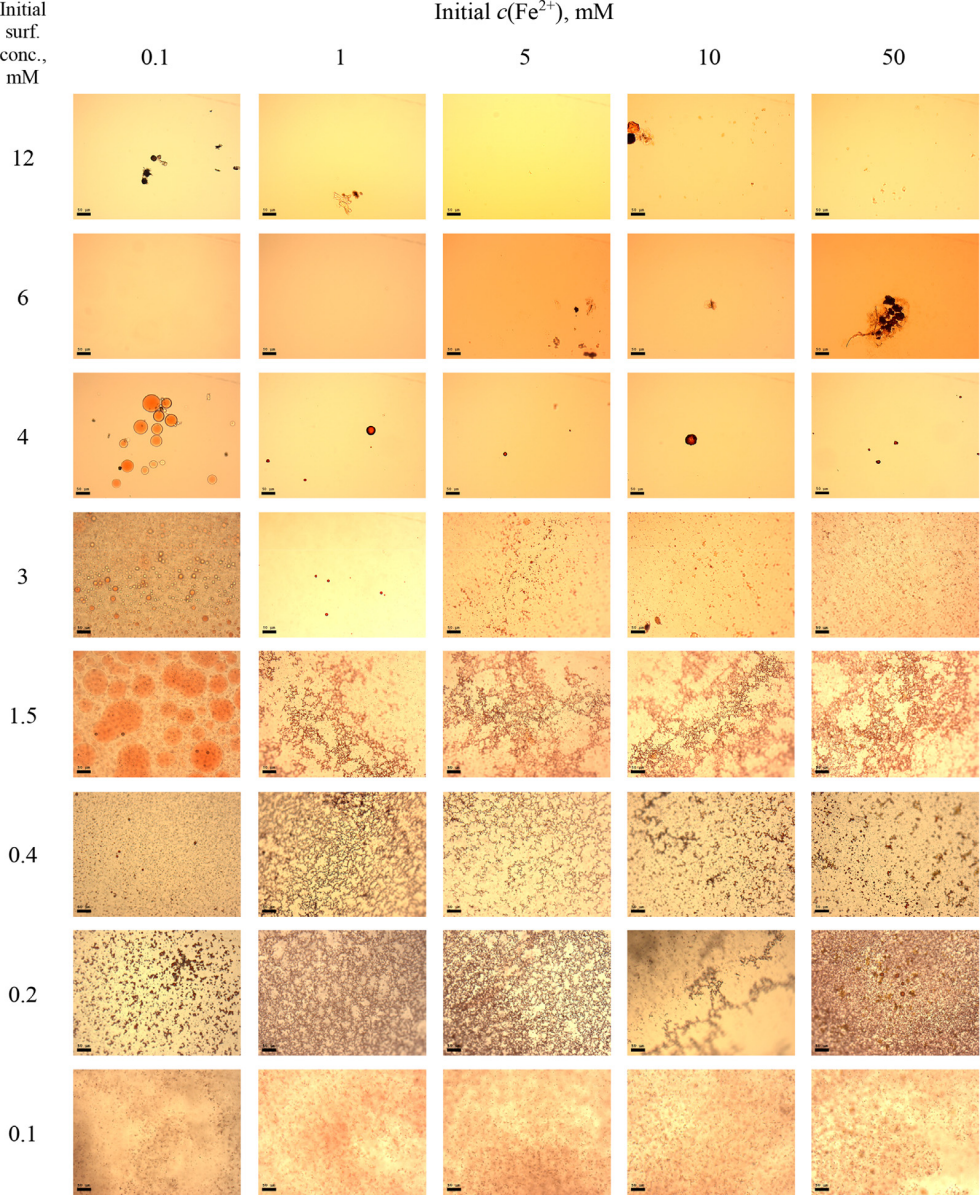
MCs based on TX-100 with initial concentrations of *c*(BPhen) = 15 mM and *c*(NaCl) = 200 mM, varying initial concentrations of the Triton and Fe^2+^ salt. Scale bar is 50 μm.

**Fig. 12:**
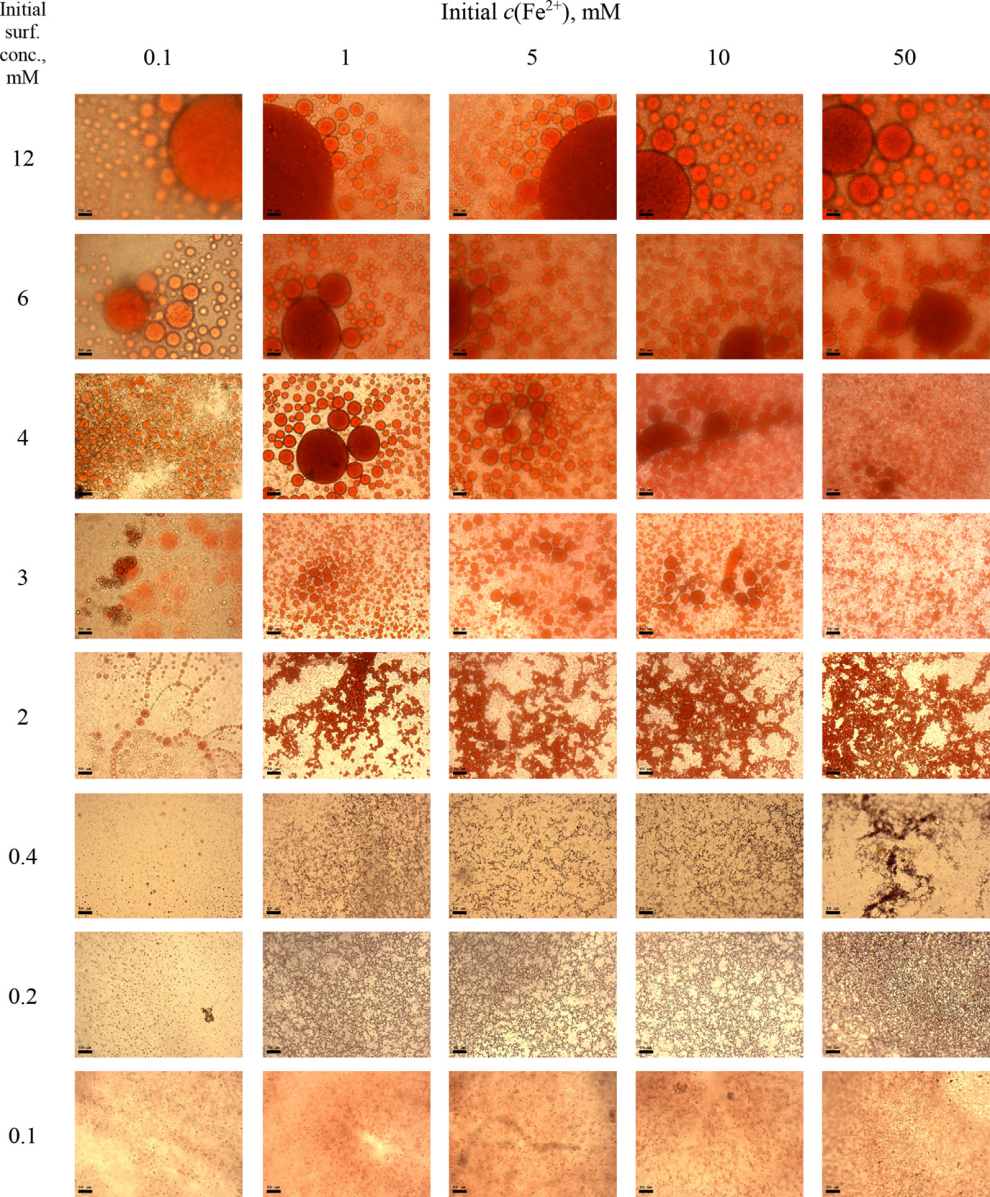
MCs based on TX-114 with initial concentrations of *c*(BPhen) = 15 mM and *c*(NaCl) = 200 mM, varying initial concentrations of the Triton and Fe^2+^ salt. Scale bar is 50 μm.

**Fig. 13:**
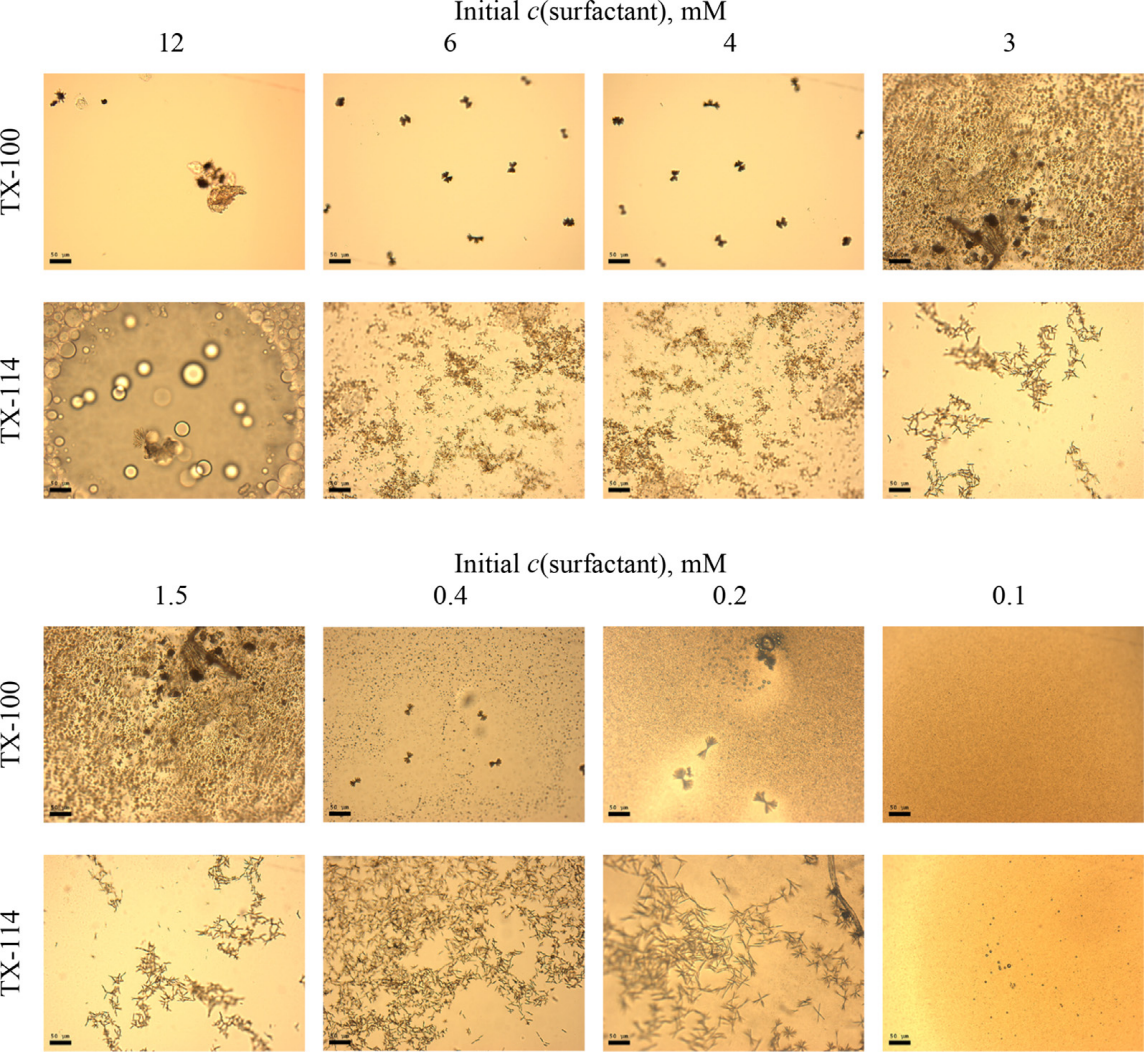
Control experiments without Fe^2+^ salt. Initial concentrations of *c*(BPhen) = 15 mM and *c*(NaCl) = 200 mM. Scale bar is 50 μm.

**Fig. 14:**
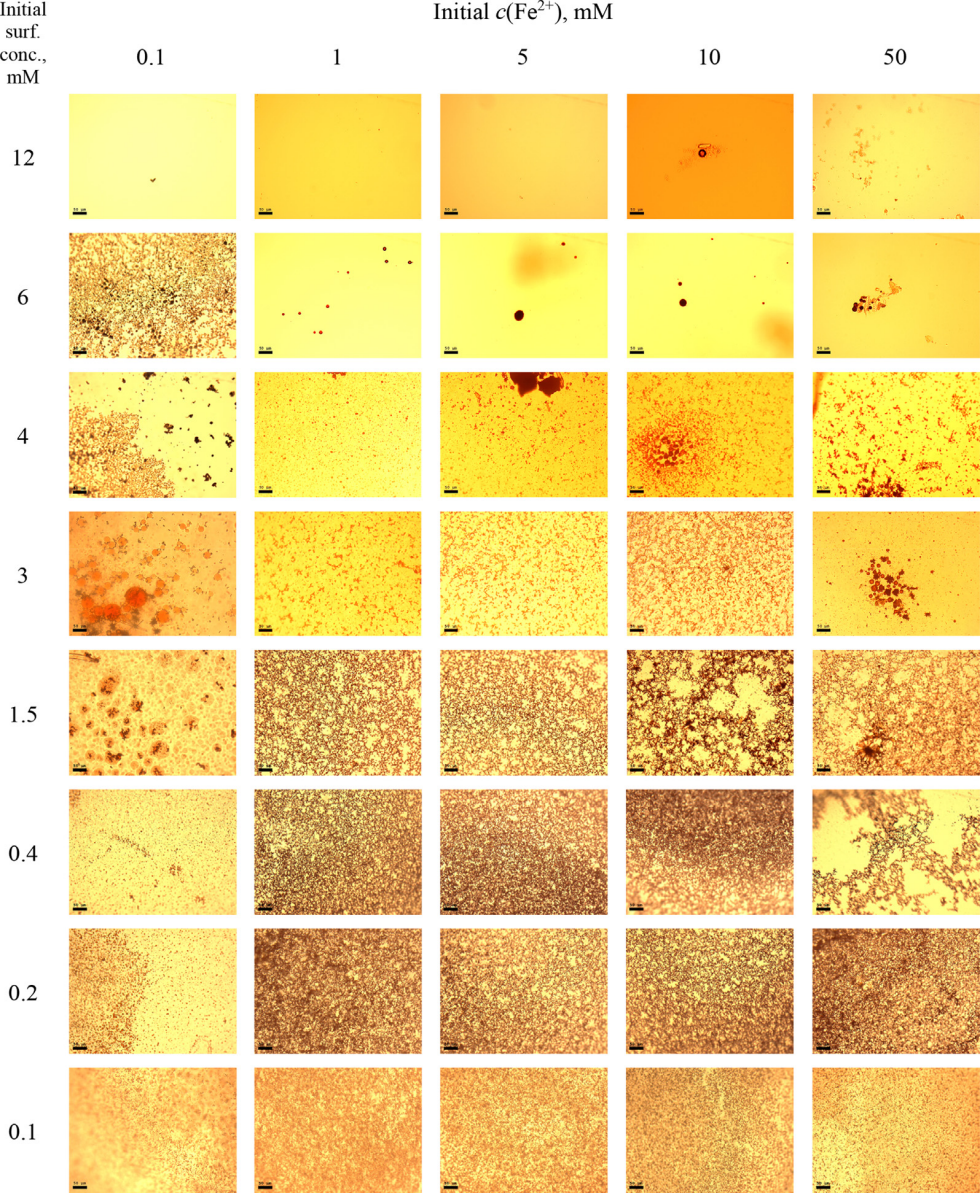
MCs based on TX-100 with initial concentrations of *c*(BPhen) = 20 mM and *c*(NaCl) = 200 mM, varying initial concentrations of the Triton and Fe^2+^ salt. Scale bar is 50 μm.

**Fig. 15:**
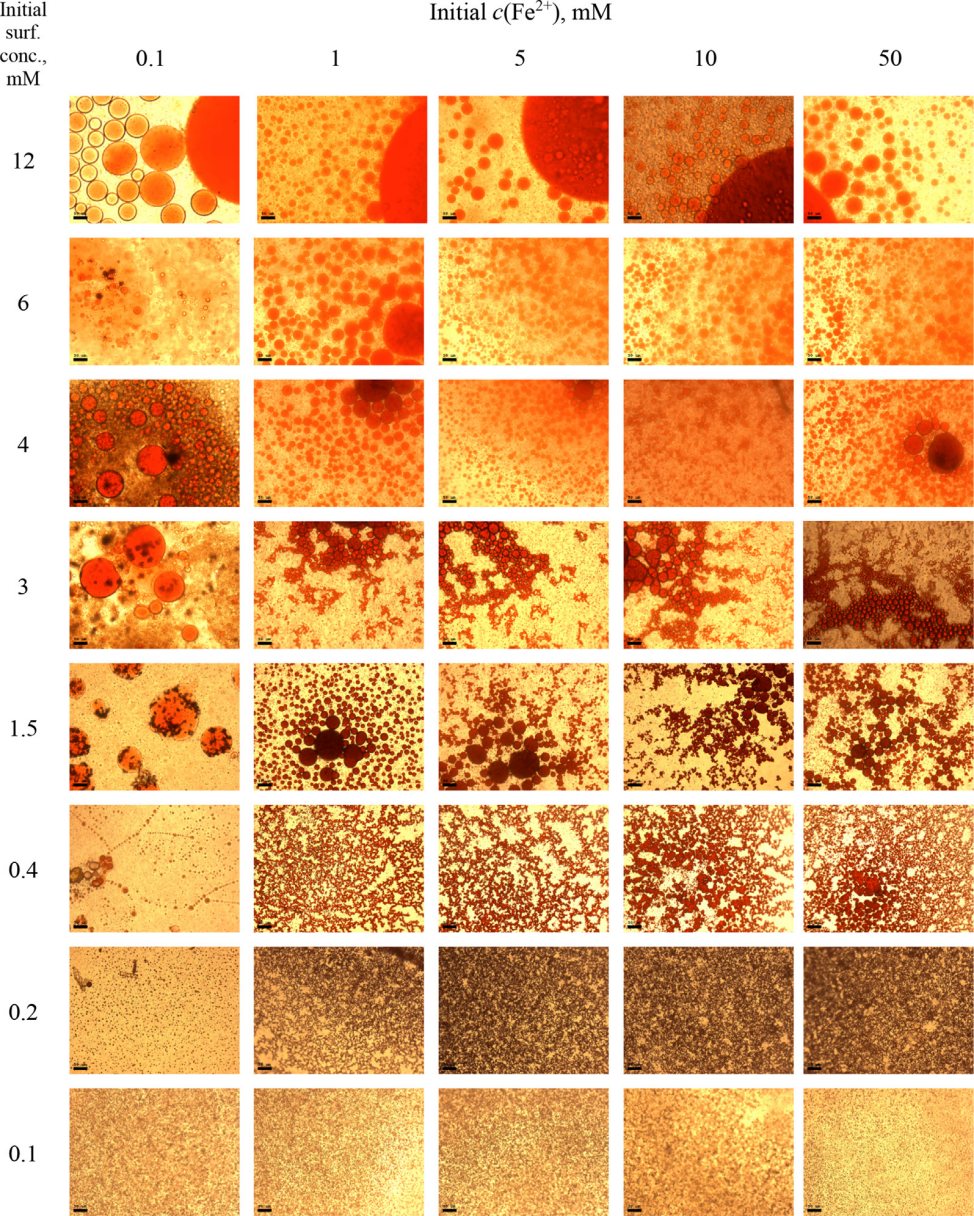
MCs based on TX-114 with initial concentrations of *c*(BPhen) = 20 mM and *c*(NaCl) = 200 mM, varying initial concentrations of the Triton and Fe^2+^ salt. Scale bar is 50 μm.

**Fig. 16:**
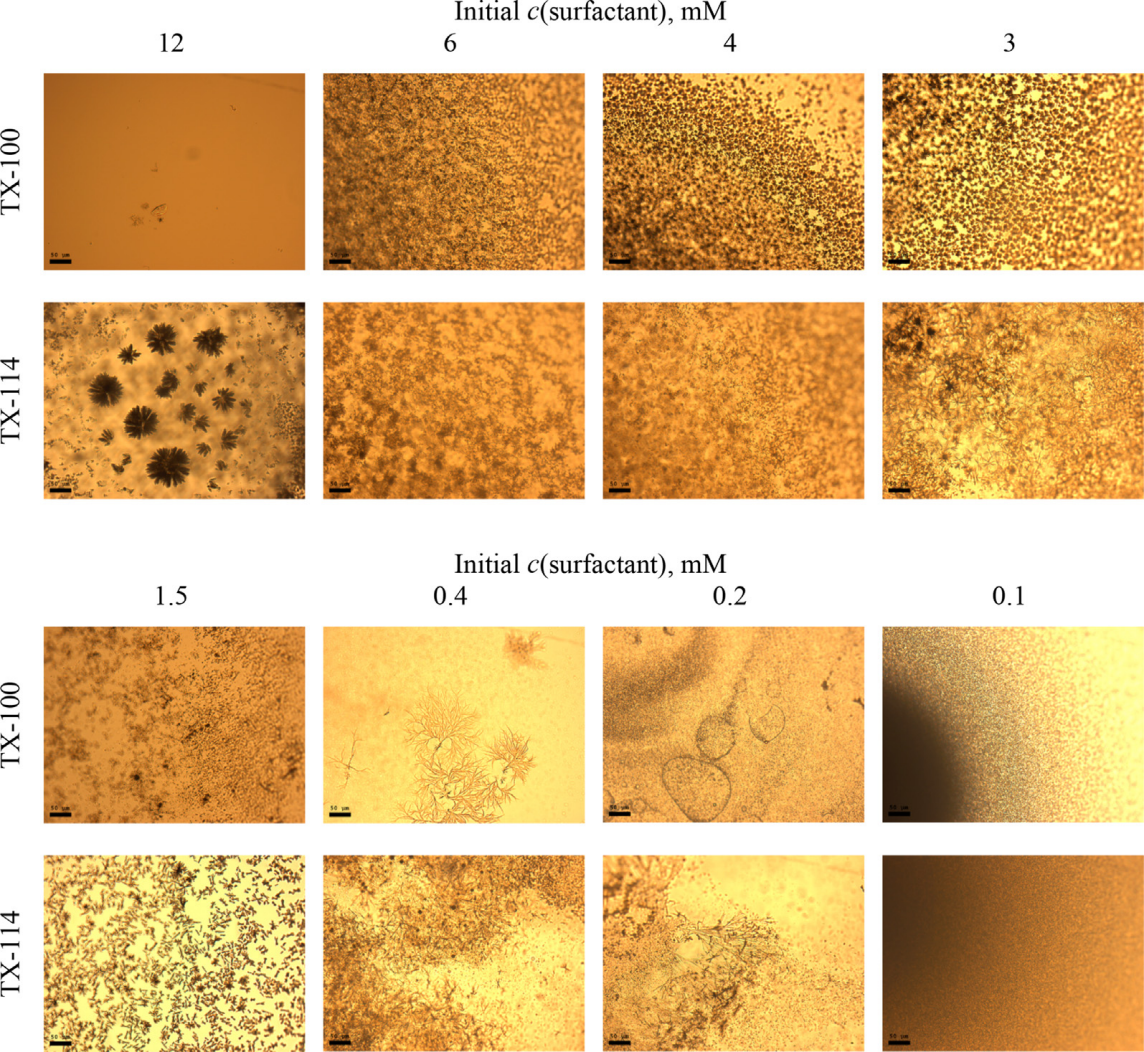
Control experiments without Fe^2+^ salt. Initial concentrations of *c*(BPhen) = 20 mM and *c*(NaCl) = 200 mM. Scale bar is 50 μm.

**Fig. 17:**
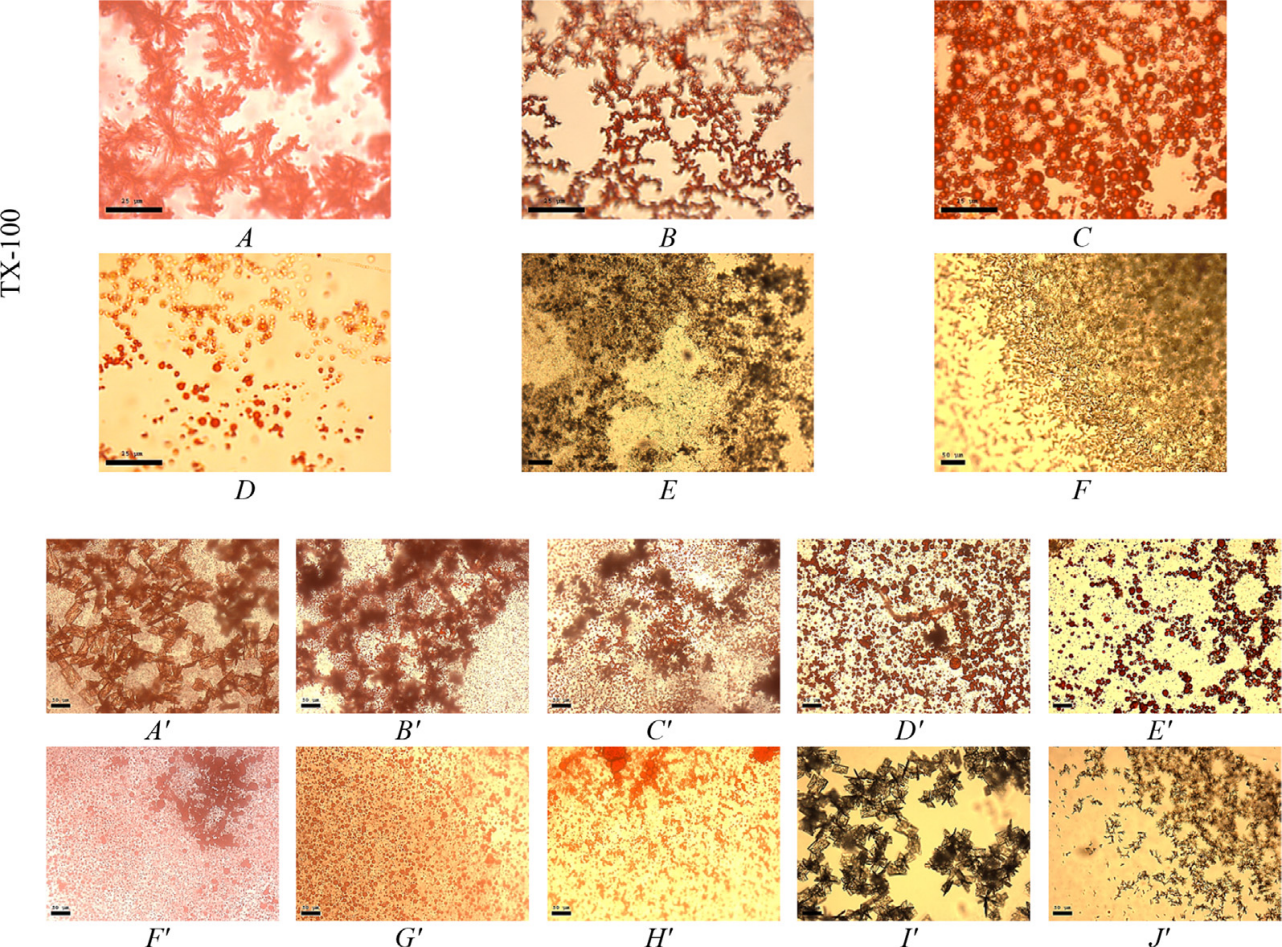
Effect of *c*_surfactant_: *c*_chelator_ ratio (*c*_M_/*c*_M_) on clustering processes during 15 min. For TX-100 from *A* to *D*: 0.60, 0.85, 1.35, 2.85 (scale bar is 25 μm), control samples (without Fe^2+^ salt), *E*, *F* for critical cases 0.50 and 2.375, respectively (scale bar is 50 μm) and for TX-114 from *A′* to *H’*: 0.064, 0.1, 0.15, 0.225, 0.35, 0.60, 1.35, 2.85, control samples (without Fe^2+^ salt), *I'*, *J'* for critical cases 0.064 and 2.85, respectively (scale bar is 50 μm).

**Fig. 18:**
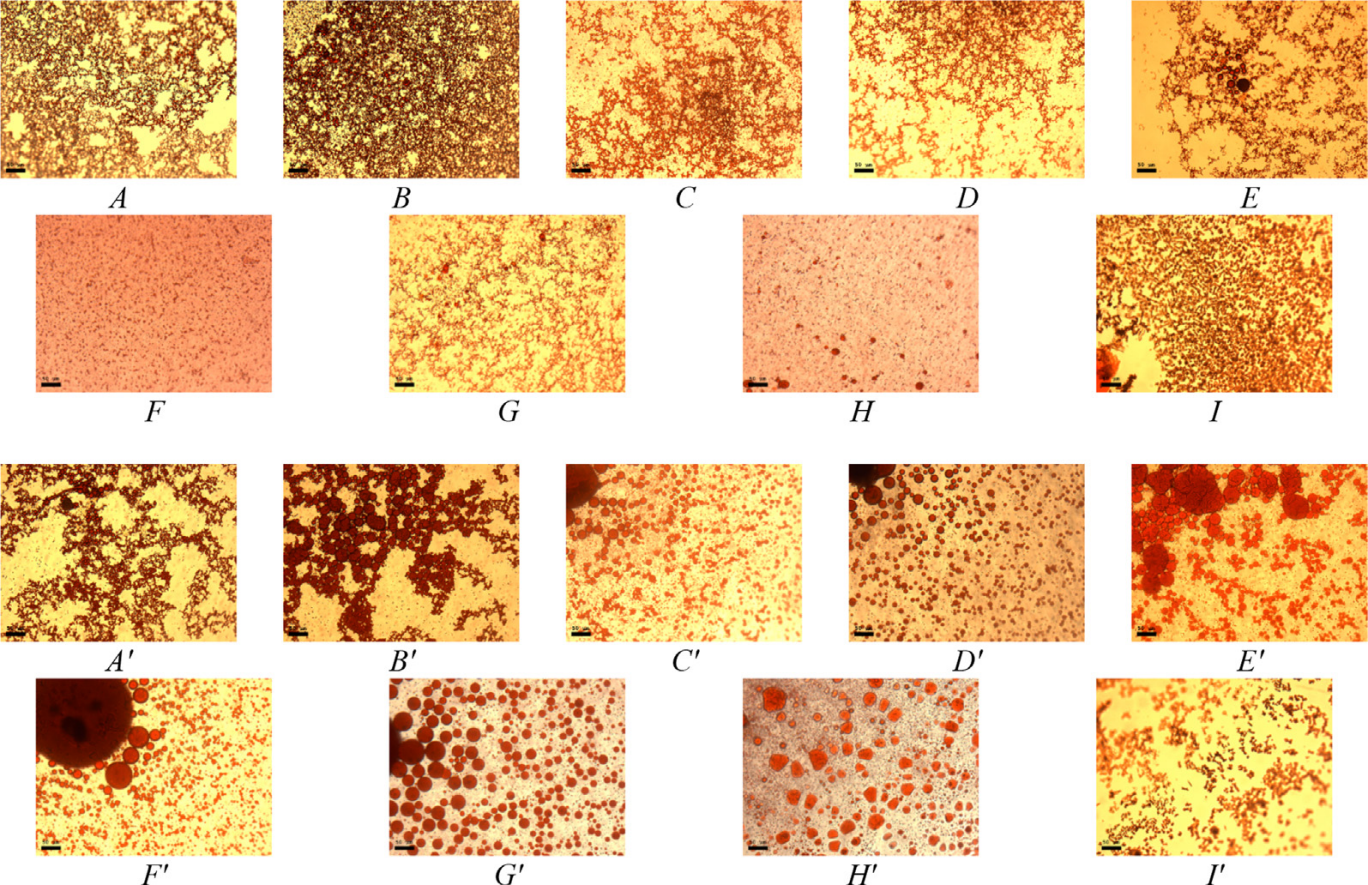
Effect of the ratio of metal ion: chelated micelles (by volume) varying on the clustering process, for TX-100/TX-114 from *A/A'* to *H/H'*: 9/1, 8/2, 7/3, 6/4, 5/5, 4/6, 3/7, 2/8, 1/9. Scale bar is 50 μm.

**Fig. 19:**
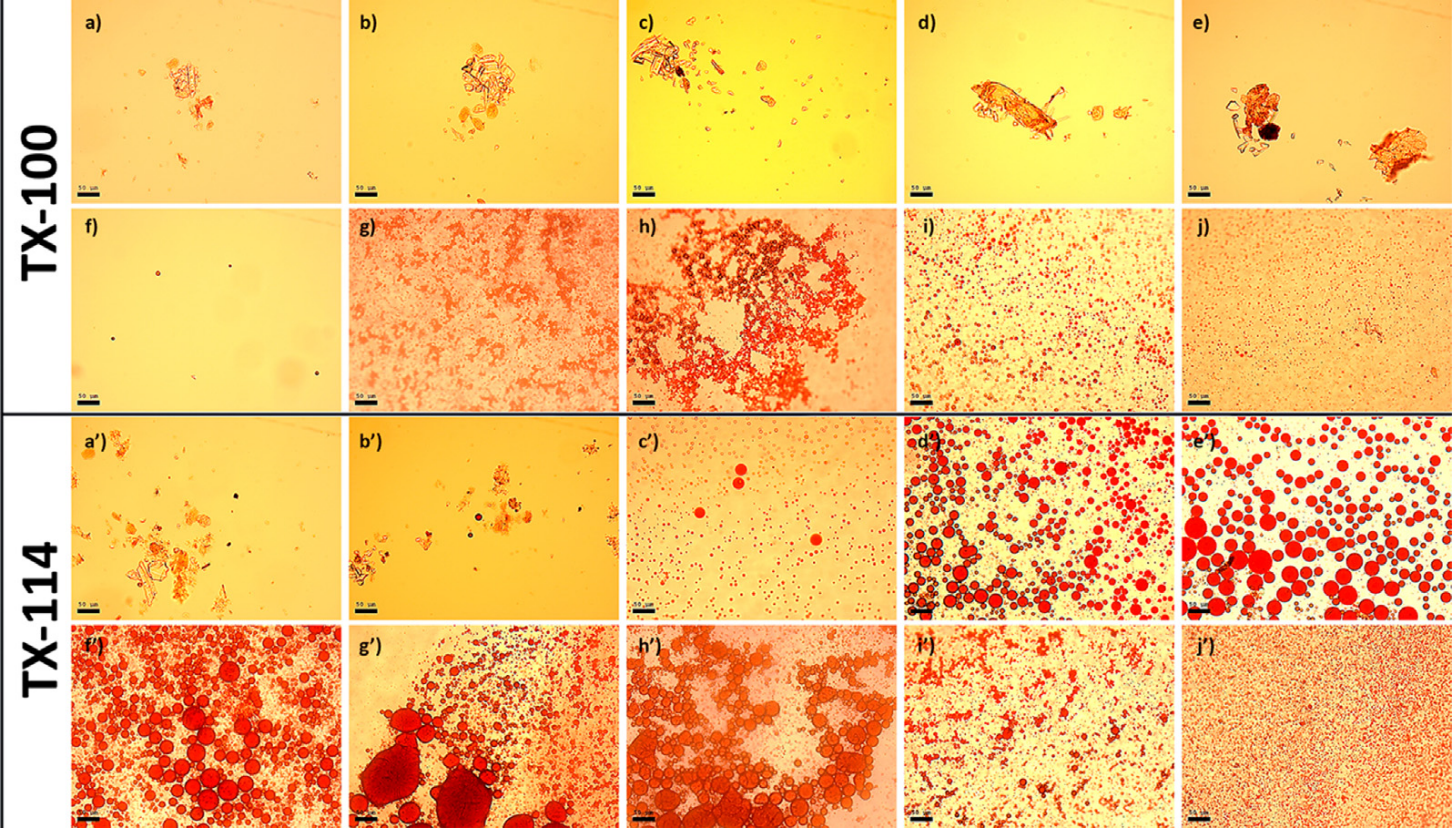
The dependence of micellar clusters formation on NaCl concertation (after 30 min), for TX-100/TX-114 from *a/a'* to *f/f'* (mM): 0, 50, 100, 150, 200, 300, 400, 800, 1000, 1600 (in accordance with General Procedure, the concentrations of all reagents were the same and stayed unchanged, while the initial concentration of NaCl was varied), scale bar is 50 μm.

**Fig. 20:**
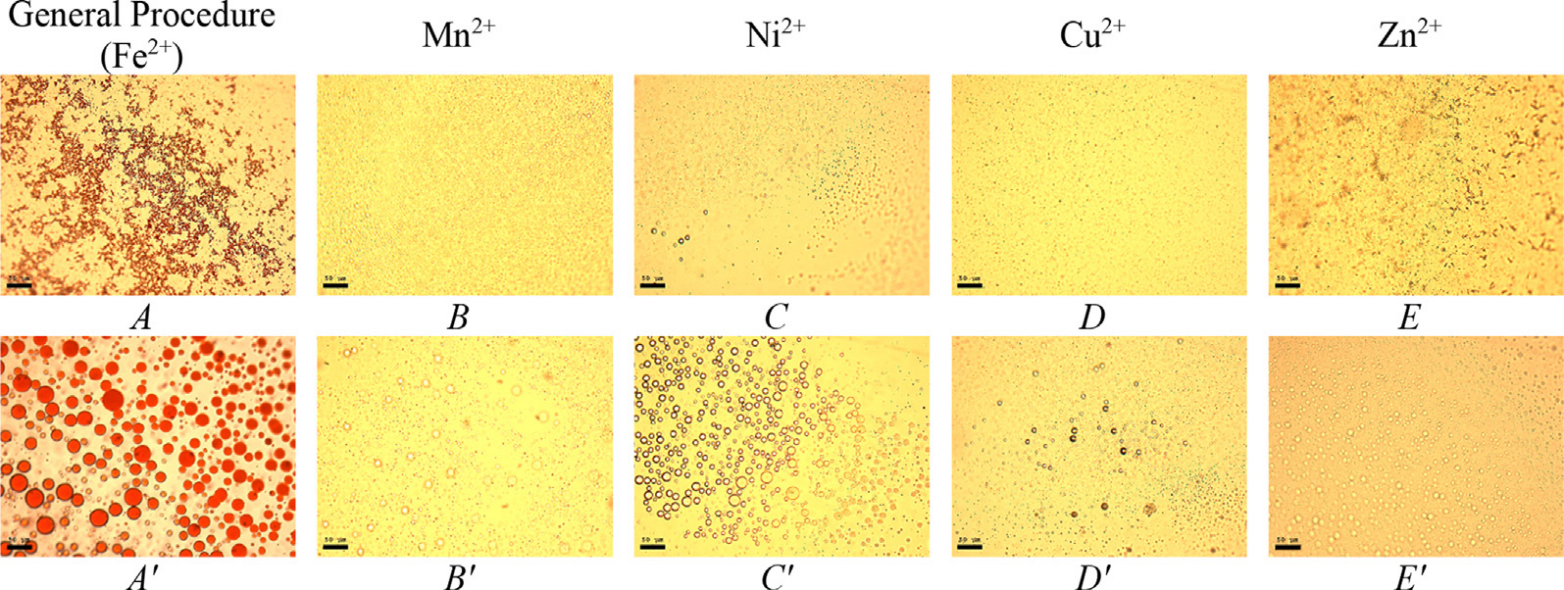
Effect of metal cations influence on micelles clusterization process. Scale bar is 50 μm.

**Fig. 21:**
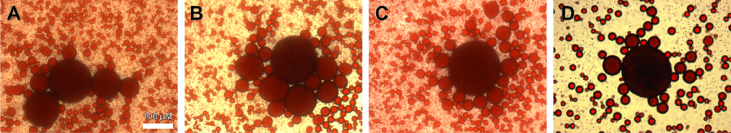
The effect of the Ni salt addition to freshly prepared Fe-based clusters. *A, C* – freshly formed MCs, *B* – after addition of 5 mM NiCl_2_ solution, *D* – after addition of 100 mM NiCl_2_ solution. Scale bar is 100 μm.

**Fig. 22:**
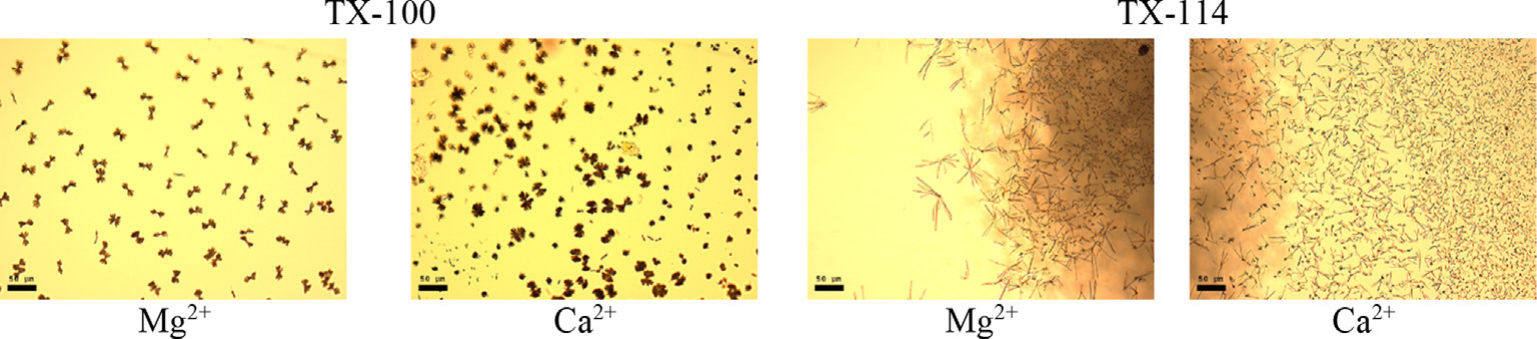
Effect of changing of *d*-metal cations onto Mg^2+^ and Ca^2+^. Scale bar is 50 μm.

**Fig. 23:**
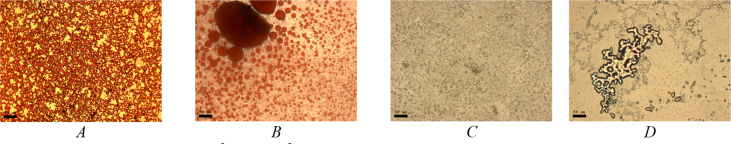
Application of Mg^2+^ and Fe^2+^ mixture (1–1 ratio, *c* = 4 mM) in clusterization process for TX-100 (*A*) and TX-114 (*B*); Effect of Fe^2+^ addition to freshly-prepared Ni^2+^-based micellar clusters, before iron salt addition (*C*) and after (*D*). Scale bar is 50 μm.

**Fig. 24:**
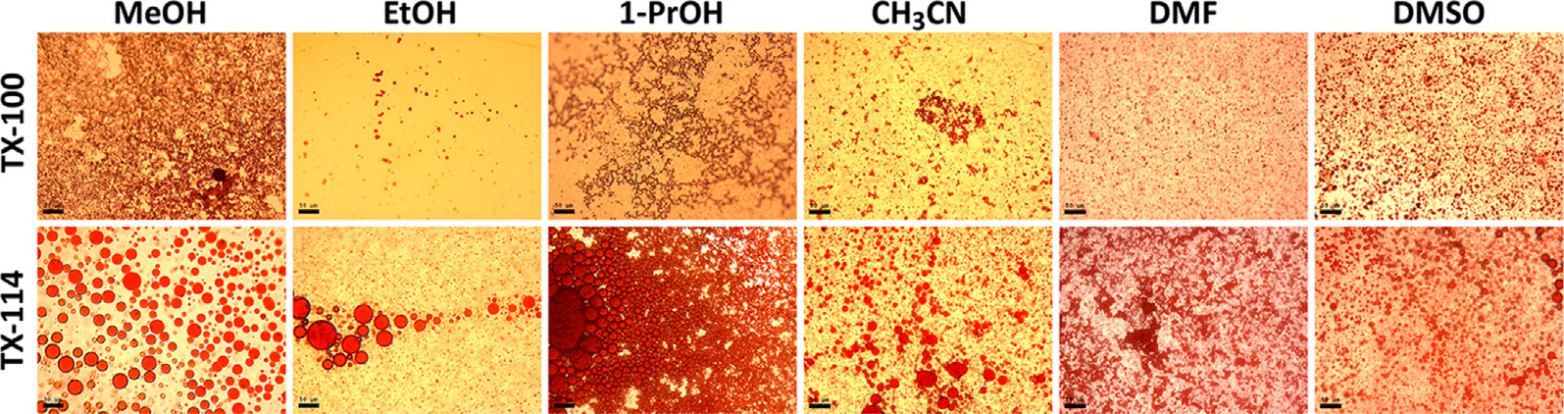
Effect of solvent replacement it which BPhen is preliminarily dissolved within equal period formation time (30 min). Scale bar is 50 μm.

**Fig. 25:**
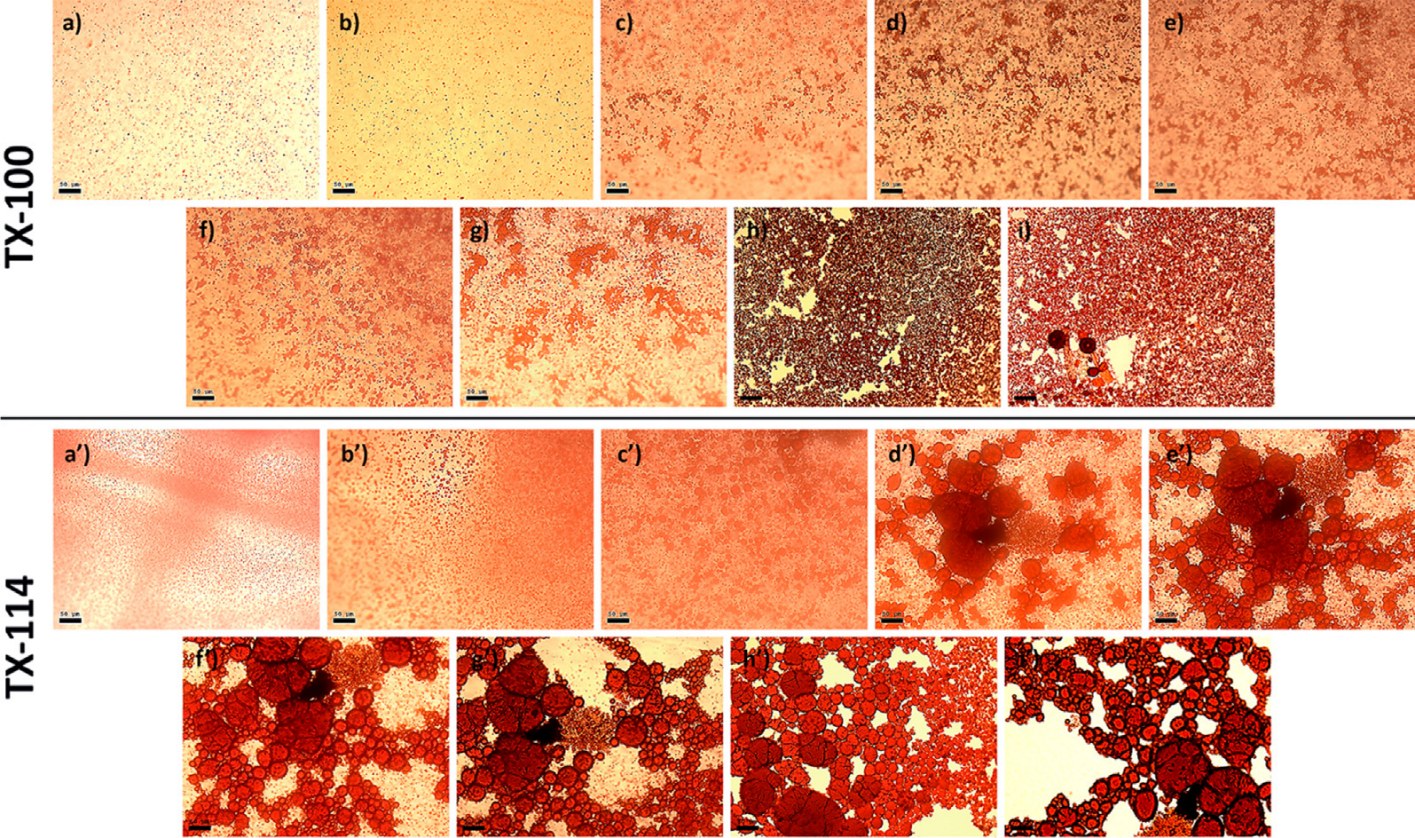
Micellar clustering kinetics, from *a/a'* to *g/g'* (min): 0, 9, 18, 27, 36, 45, 54; *h/h'* – 48 h; *i/i'* – 7 days. Scale bar is 50 μm.

**Fig. 26:**
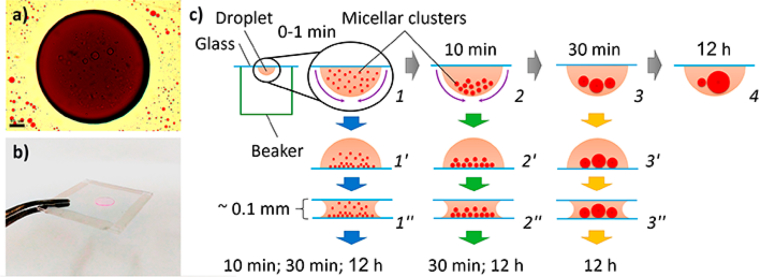
A sample of huge micellar cluster (*a*), demonstration of modified clusters preparation procedure (*b*), an application of modified procedure regarding clusters size varying (Sequences 1-1′-1''; 1-2-2′-2″ and 1-2-3-3′-3″) and comparison with General Procedure, sequence 1-2-3-4 (*c*). Scale bar is 50 μm.

**Fig. 27:**
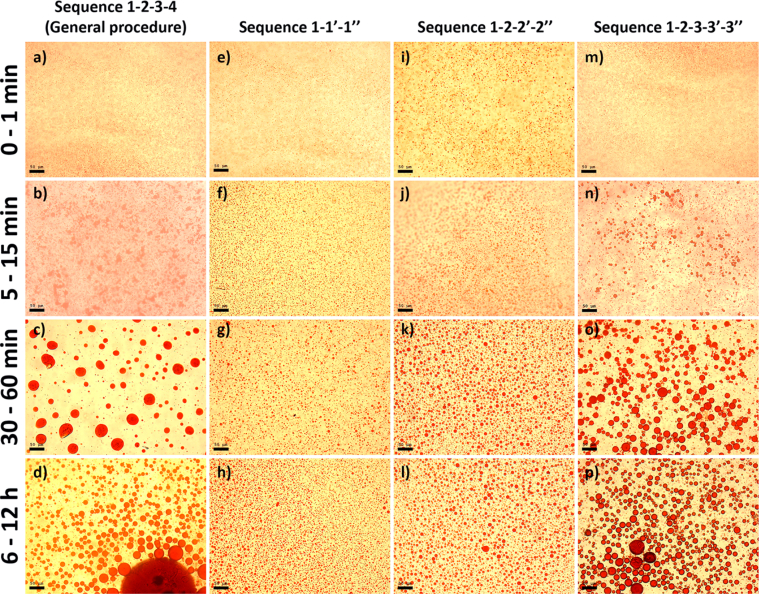
Comparison between clusters size obtained by General Procedure depending on time (*a – d*) with the modified procedure (*e*–*p*) according to the scheme shown in Fig. 26. Scale bar is 50 μm.

**Fig. 28:**
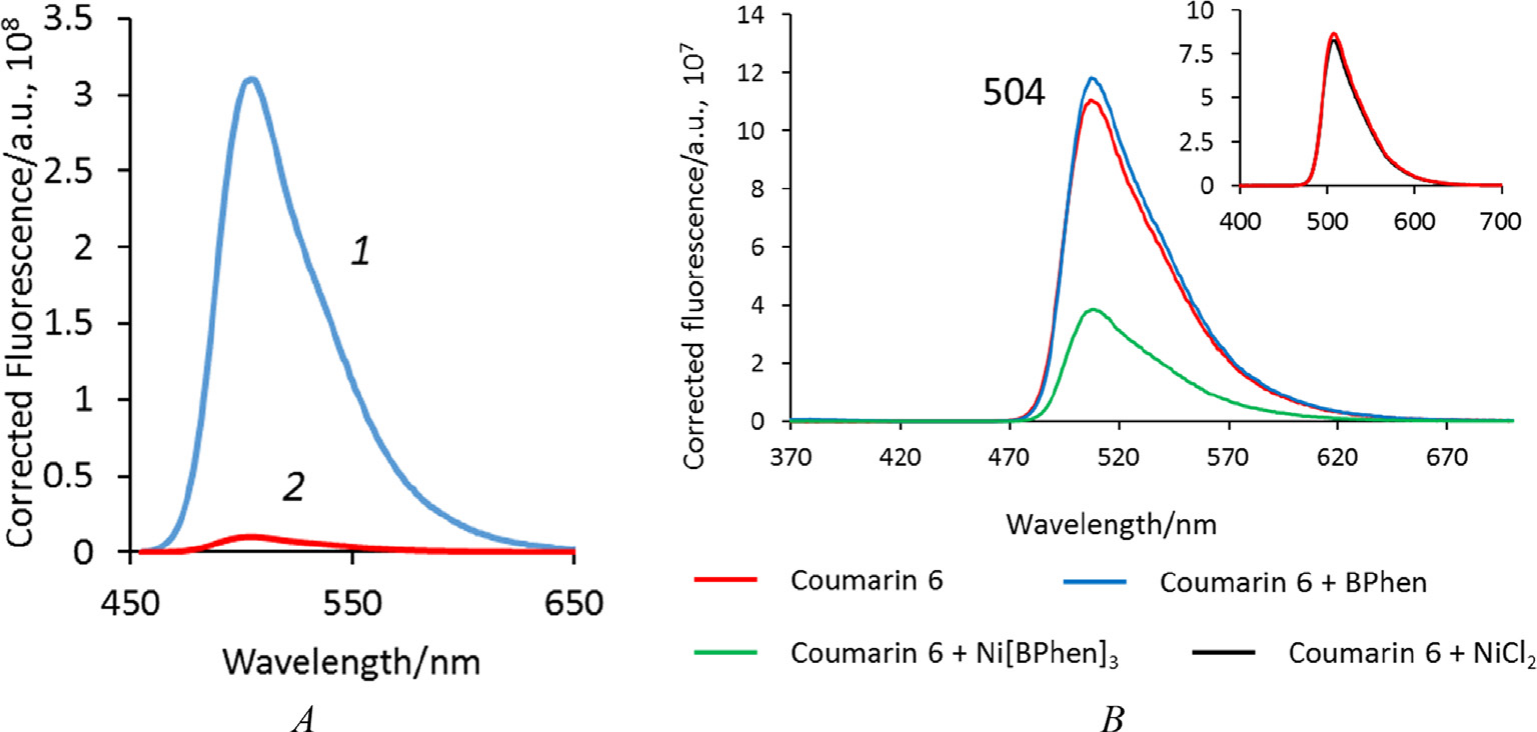
(*A*) Fluorescence spectrum of MCs emulsion of Coumarin 6 (C6) based on support compound scheme (Triton/BPhen/C6) after 30 min of formation in 1 mm thickness cuvette (*1*) and a spectrum of the same solution after 12 h of incubation followed by MCs separation (*2*); (*B*) Effect of fluorescence quenching of Coumarin 6 by [Ni[BPhen]_3_]^2+^. Inset: control experiment with Ni^2+^ salt addition. Final concentrations of all reagents in solution were ∼1·10^−5^ M; measurement parameters: λ_ex_ = 365 nm, ex. and em. slits were 1 nm, quartz cuvette, 1 cm thickness.

**Scheme 1:**
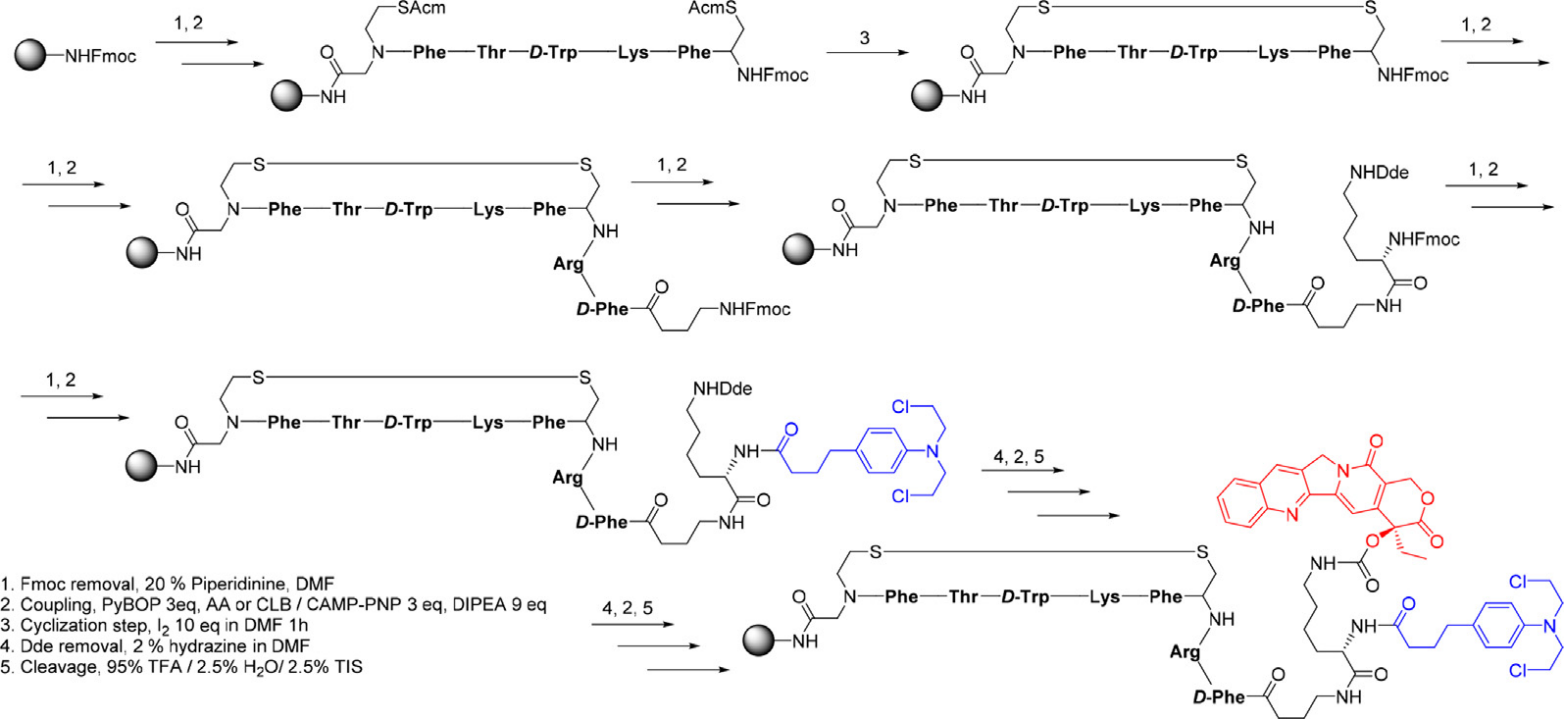
Scheme of anticancer drug synthesis.

**Fig. 29:**
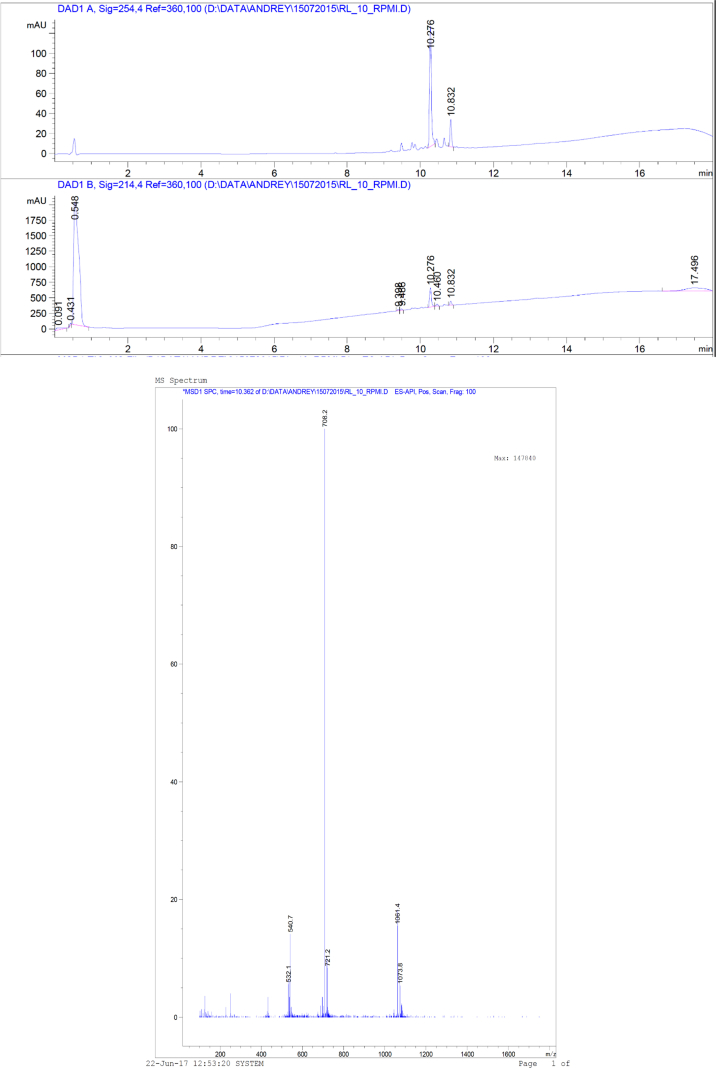
HPLC and LCMS chromatograms of PTR-58-CLB-CAMP.

**Fig. 30:**
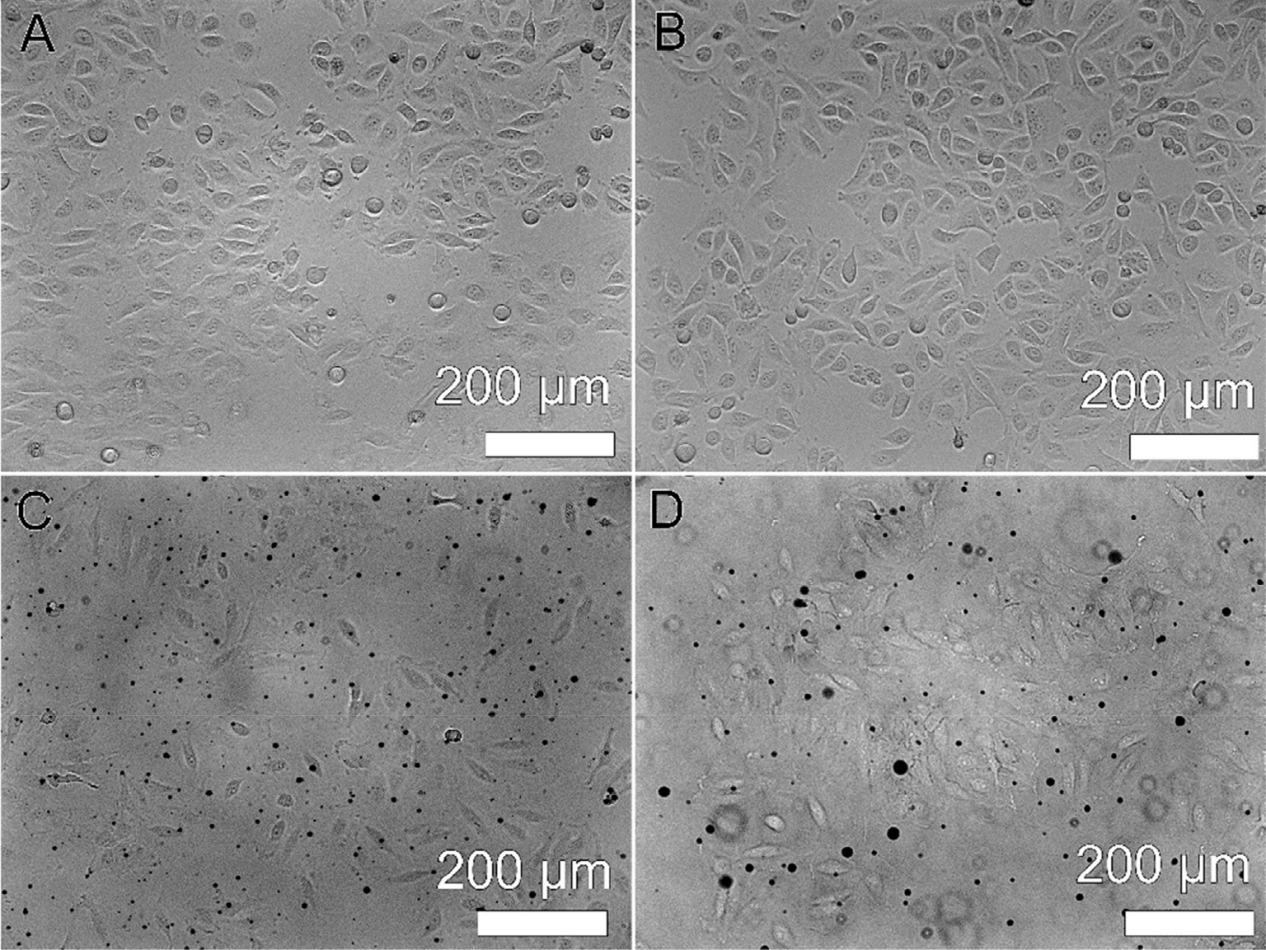
Bright field optical microscopy images of HeLa cells as-grown (*A*) and immediately after the infusion by 200 μL Triton-family-based MCs (*C*), and after 24 h incubation without (*B*) and with (*D*) infused clusters. Cells viability histogram is presented in the supporting information file (Fig. S1).

This article includes the General Procedure to synthesize micellar clusters (MCs) based on the Triton-X family surfactants (TX-100 and TX-114). Application of the General Procedure and the effect of numerous alterations in the solution composition to prepare various MCs are presented in Fig. 1, Fig. 2, Fig. 3 and Table 1. Raw images measured by optical microscopy of the varying effect of metal ion, surfactant, and chelator concentration are shown in Fig. 4, Fig. 5, Fig. 6, Fig. 7, Fig. 8, Fig. 9, Fig. 10, Fig. 11, Fig. 12, Fig. 13, Fig. 14, Fig. 15, Fig. 16, the effect of surfactant-chelator relation change is shown in Table 2 and Fig. 17, while metal ion-micelle concertation ratio variation is presented in Table 3 and Fig. 18, Fig. 19. The effect of metal ions replacement by co-chelator on the MCs formation is demonstrated in Fig. 20, Fig. 21, Fig. 22, Fig. 23 and the effect of solvent and chelator replacement is shown in Fig. 24 and Table 4. Kinetics of the MCs formation is presented in Fig. 25, Fig. 26, Fig. 27. The solubilization of fluorescent dye such as Coumarin 6 in the MCs is shown in Fig. 28. The scheme of PTR-58-CLB-CAMP synthesis (Scheme 1), HPLC and LCMS chromatograms of the drug are presented in Fig. 29 and Table 5, Table 6. Finally, the viability of HeLa cells with and without the presence of drug-free Triton-X-based family MCs is shown in Fig. 30. A summary table describing the effect of TX-100 and TX-114 applying in the micellar clustering and the most useful parameters of the MCs as well as additional data of the cells viability when treating with MCs are presented in the supplementary file (Scheme S1).

**Table 1:** Various synthesis procedures for check the Triton micelles clusterization.

	Scheme	[A]	[B]	[C]	[D]
1.^a^		3 mM of surfactant in TDW	20 mM BPhen in MeOH	2 M NaCl in TDW	100 mM FeSO_4_ in TDW
2.		3 mM of surfactant in TDW	20 mM BPhen in MeOH	TDW	100 mM FeSO_4_ in TDW
3.		3 mM of surfactant in TDW	MeOH	TDW	100 mM FeSO in TDW
4.		3 mM of surfactant in TDW	MeOH	2 M NaCl in TDW	100 mM FeSO_4_ in TDW
5.		3 mM of surfactant TDW	MeOH	2 M NaCl in TDW	TDW
6.		3 mM of surfactant in TDW	20 mM BPhen in MeOH	2 M NaCl in TDW	TDW
7.		3 mM of surfactant in TDW	20 mM BPhen in MeOH	TDW	TDW
8.		TDW	20 mM BPhen in MeOH	2 M NaCl in TDW	100 mM FeSO_4_ in TDW
9.		TDW	20 mM BPhen in MeOH	TDW	100 mM FeSO_4_ in TDW
10.		TDW	20 mM BPhen in MeOH	2 M NaCl in TDW	TDW
11.		TDW	MeOH	2 M NaCl in TDW	100 mM FeSO_4_ in TDW

– Triton X Micelle;  – BPhen chelator;  – Mfx1et al. ion;  – Ambient electrolyte (NaCl).

**For increased BPhen concentration checking, in schemes 8, 9 and 10, Solution 1 contained 20 mM BPhen in MeOH only.

a General Procedure.

**Table 2:** Surfactant-chelator relation change.

Before Fe^2+^ adding (in chelated micelles)				After Fe^2+^ adding (in micellar clusters)	
Volume ratio, *V*_*surfactant*_*:V*_*chelator*_*(μl/μl)*	*c*_*surfactant*_*, mM*	*c*_*chelator*_*, mM*	Concentration ratio*, c*_*surfactant:*_*c*_*chelator*_*(mM/mM)*	*c*_*surfactant*_*, mM*	*c*_*chelator*_*, mM*
95/5	2.85	1	2.85	1.425	0.5
90/10*	2.70	2	1.35	1.35	1
85/15	2.55	3	0.85	1.275	1.5
80/20	2.40	4	0.60	1.20	2
70/30	2.10	6	0.35	1.05	3
60/40	1.80	8	0.225	0.90	4
50/50	1.50	10	0.15	0.75	5
40/60	1.20	12	0.10	0.60	6
30/70	0.90	14	0.064	0.45	7

*Corresponds to General Procedure.

**Table 3:** Metal ion-micelles concertation ratio variation.

Volume ratio, V_chelated micelles solution_:V_Fe_^2+^_salt_ (*μ*l/*μ*l)	Final concentrations in micellar clusters			Final concentration ratio in micellar clusters	
	*c*_surfactant_, mM	*c*_chelator_, mM	*c*_Fe_^2+^_salt_, mM	*c*_surfactant:_*c*_Fe_^2+^_salt_ (mM/mM)	*c*_chelator_: *c*_Fe_^2+^_salt_ (mM/mM)
1/9	0.27	0.2	1.8	0.15	0.11
2/8	0.54	0.4	1.6	0.34	0.25
3/7	0.81	0.6	1.4	0.58	0.43
4/6	1.08	0.8	1.2	0.90	0.06
5/5*	1.35	1	1	1.35	1.00
6/4	1.62	1.2	0.8	2.03	1.50
7/3	1.89	1.4	0.6	3.15	2.33
8/2	2.16	1.6	0.4	5.40	4.00
9/1	2.43	1.8	0.2	12.2	9.00

*Corresponds to the General Procedure.

**Table 4:** Solvents parameters.

	MeOH	EtOH	1-PrOH	CH_3_CN	DMF	DMSO
Boiling point temperature (BP, K)	337.8	351.6	370.6	354.5	426.2	462.2
Vapor pressure density (VP, kPa, at 293.15 K)	12.8	5.8	1.9	9.7	0.5	0.06
Dynamic viscosity (*ν*, mPa·s, at 298.15 K)	0.55	1.07	1.96	0.39	0.80	1.99
Dipole moments (*μ*)	1.71	1.69	1.68	3.93	3.82	3.96

**Table 5:** High-Performance Liquid Chromatography (HPLC) measurements. Semi preparative gradient.

Time	Mobile phase A(%)	Mobile phase B(%)
0	100	0
3	100	0
7	75	25
37	25	75
40	0	100
45	0	100
46	100	0
50	100	0

**Table 6:** Liquid Chromatography Mass Spectrometry (LCMS) measurements.HPLC gradient.

Time	Mobile phase A(%)	Mobile phase B(%)
0	100	0
3	100	0
8	0	100
13	0	100
15	100	0
17	100	0

### 1.1 Application of the General Procedure for clusterization of triton micelles

To prove that the TX-100 and TX-114 clusters formation mechanism is specific and requires simultaneous presence all of four components in the mixture, according to the General Procedure, we have attempted to remove one or two of the components of the mixture. Therefore, the variations in the preparation procedure may be presented schematically as follows for each scheme in Table 1. Solution 1: 45 μl of [A] + 5 μl of [B], vortex 5 sec; solution 2: 760 μl of TDW + 200 μl of [C] + 40 μl of [D], vortex 5 sec. Droplets preparation: add 5 μl of solution 2–5 μl of solution 1. The data are presented on Fig. 1, Fig. 2, Fig. 3.

### 1.2 Varying of metal ion, surfactant, and chelator concentration

In the general procedure, initial concentrations of all reagents were varied, while the concentration of NaCl was constant. The data are presented on Fig. 4, Fig. 5, Fig. 6, Fig. 7, Fig. 8, Fig. 9, Fig. 10, Fig. 11, Fig. 12, Fig. 13, Fig. 14, Fig. 15, Fig. 16.

### 1.3 Surfactant-chelator relation change

Initial concentrations, mM, *c*(surfactant) = 3; *c*(chelator) = 20; *c*(FeSO_4_) = 2; *c*(NaCl) = 400. The data are presented in Table 2 and Fig. 17.

### 1.4 Metal ion-micelles concertation ratio variation

Initial concentrations, mM, *c*(surfactant) = 3; *c*(chelator) = 20; *c*(FeSO_4_) = 2; *c*(NaCl) = 400; Chelator-micelles aggregate solution (100 μl, *c*_surfactant_ = 2.7 mM, *c*_chelator_ = 2 mM, ratio = 1.35): 45 μl 3 mM surfactant + 5 μl 20 mM chelator. The data are presented in Table 3 and in Fig. 18, Fig. 19.

### 1.5 Effect of metal ion replacement

Fe^2+^ salt in the General Procedure was replaced by the salts, containing appropriate metal ion (NiCl_2_, MnCl_2_, CuCl_2_, ZnCl_2_, MgCl_2_, CaCl_2_) or mixture. Initial salt concentration and concentrations of other components remained the same as in the case of the General Procedure. The data are presented in Fig. 20, Fig. 21, Fig. 22, Fig. 23.

### 1.6 Effect of solvent replacement

The data are presented in Fig. 24 and Table 4.

### 1.7 Kinetics of clusters formation

The data are presented in Fig. 25, Fig. 26, Fig. 27.

### 1.8 Fluorescent dye solubilization

A saturated dye solution of Coumarin 6 was prepared by dissolving it in MeOH. The prepared solution is vortexed vigorously for 5 min followed by centrifugation for 1 min at 5000 rpm and removing of undissolved substance. The concentration of the dye in the saturated solution was about 15 mM.

For encapsulation of the dye, several routes are possible. The first one is to add 1 μl of desired dye to 10 μl drop, containing freshly prepared micellar clusters, obtained by General Procedure, based on Ni-BPhen complex. The second is to add 5 μl–50 μl of freshly prepared micelle-chelator complex, to take 5 μl of the solution and to mix it with 5 μl of the salts solution (solution 2), as in the case of the General Procedure. The third route is to add 1 μl of desired dye to 5 or 10 μl of pure surfactant or 1 μl–5 μl (or 10 μl, with NaCl) of micelle-chelator aggregate (‘Support compound Scheme’), followed by incubation of the drops at 18–20 °C over a reservoir sealed with silicon grease (24 well tissue culture plate VDX (Hampton Research) or Corning Inc.) containing 0.5 ml 200 mM NaCl or H_2_O. The data are presented in Fig. 28.

Summary of the most useful parameters for Triton-X-based MCs synthesis and followed encapsulation of the peptide anticancer drugs and hydrophobic compounds is presented in the supporting file (Table S1).

### 1.9 Anticancer drug synthesis

The data are presented in Scheme 1, Fig. 29 and Table 5, Table 6.

### 1.10 The viability of HeLa cells with and without the presence of drug-free triton-x-based family MCs

The data are presented in Fig. 30 and in Fig. S1 (supporting information).

## 2 Experimental design, materials, and methods

### 2.1 Materials

Polyethylene glycol *tert*-octylphenyl ethers, Triton TX-100 (M_avg_ = 625 g/mol, for molecular biology) and TX-114 (M_avg_ = 537 g/mol, laboratory grade); 7-diphenyl-1,10-phenanthroline (bathophenantroline, BPhen, ≥ 99%), FeSO_4_ (heptahydrate, ACS reagent, ≥ 99%); NaCl (BioXtra, ≥ 99.5%); NiCl_2_ (anhydrous, 98%); MnCl_2_ (anhydrous, ≥99% trace metals basis); CuCl_2_ (anhydrous, 99%); ZnCl_2_ (anhydrous, reagent grade, ≥98%); MgCl_2_ (anhydrous, ≥98%); CaCl_2_ (anhydrous, ACS reagent, ≥96%); Coumarin 6 (≥99%); MeOH (anhydrous, 99.8%), EtOH (puriss. p.a., absolute, ≥99.8%), 1-PrOH (anhydrous, 99.7%), DMF (anhydrous, 99.8%); DMSO (≥99%); CH_3_CN (anhydrous, 99.8%), “Chimmed” (Russia) and used without additional purification. For all experiments, triply-distilled deionized metal-free water (TDW) was used.

### 2.2 Synthesis

#### 2.2.1 The General Procedure of MCs droplets synthesis

The General Procedure of the Triton-X based MCs formation procedures is described in [1] and is based on the works [2], [3], [4], [5], [6], [7].

#### 2.2.2 Anticancer peptide drug conjugates synthesis

Camptothecin *(*CAMP), chlorambucil (CLB), all protected amino acids, resin, and coupling reagents were purchased from Tzamal D-Chem Laboratories Ltd. Petah-Tikva, Israel. All the solvents were purchased from Bio-Lab Ltd. Jerusalem, Israel or Gas Technologies Ltd., Kfar-Saba, Israel.

*Synthesis of Peptide-Drug conjugates:* The synthesis of the cyclic peptide was done by following a previously described procedure [8], [9], [10], [11], [12]. Briefly, in a reaction vessel equipped with a sintered glass bottom, ring amide MBHA (4-(2′,4′-Dimethoxyphenyl-Fmoc-aminomethyl)-phenoxyacetamidomethylbenzhydryl) resin, (substitution level 0.56 mmol/g, 1 g) was swelled in NMP (N-methylpyrrolidone) by agitation overnight. The Fmoc group was removed from the resin by treatment with 20% piperidine in DMF (10ml) for 10 min. This action was repeated twice. After washing the resin with NMP (7 times, 10 ml, 2 min each time), Fmoc-GlyS_2_(Acm)-OH (N-(((9H-fluoren-9-yl)methoxy)carbonyl)-N-(2-((acetamidomethyl)thio)ethyl)glycine) building unit 31 (3 eq, 10.5 mmol, 0.64 g) dissolved in NMP (7 ml) was activated with PyBoP (3 eq, 10.5 mmol, 0.7 g) and DIPEA (N, N-Diisopropylethylamine, 6 eq, 21 mmol, 0.521 ml) for 4 min at room temperature, transferred to the reaction vessel and allowed to react for 1 h at rt. Following coupling, the peptidyl-resin was washed with NMP (5 times, 7 ml, 2 min each time). Completion of the reaction was monitored by ninhydrin test (Kaiser test, yellow). Linear peptide was synthesized under standard Fmoc protocol, with 3 equivalents of each amino acid and 3 equivalents of PyBop as a coupling reagent. The deblock mixture was 80:20 DMF/piperidine (v/v).

*Cyclization step.* After coupling of Fmoc-Cys(Acm)-OH and NMP wash, the resin was washed with 4:1 DMF/water (3 times, 6.5 ml, 2 min each time). A solution of I_2_ (10 eq, 35 mmol, 1.29 g) in 4:1 DMF/water (10 ml) was added to the peptidyl – resin followed by agitation at rt for 1h to afford the disulfide bridge cyclization. The peptidyl-resin was filtered and washed extensively with 4:1 DMF/water (7 times, 10 ml, 2 min each time), DMF (6 times, 10 ml, 2 min each time), DCM (Dichloromethane) (6 times, 10 ml, 2 min each time), CHCl_3_ (4 times, 10 ml, 2 min each time), 2% ascorbic acid in DMF (6 times, 10 ml, 2 min each time) and last wash with DMF (6 times, 10 ml, 2 min each time). Finally, the coupling of last amino acid Fmoc-*D*-Phe-OH after cyclization, give cyclic peptide.

*Coupling of Fmoc-g-aminobutyric acid (linker).* Fmoc-γ-aminobutyric acid (3 eq, 10.5 mmol, 0.49 g) dissolved in NMP (7 ml) was activated with PyBoP ((Benzotriazol-1-yl-oxy)tripyrrolidinophosphonium hexafluorophosphate) (3 eq, 10.5 mmol, 0.7 g) and DIPEA (6 eq, 21 mmol, 0.521 ml) for 4 min at room temperature, transferred to the reaction vessel and allowed to react for 1h at rt. After post coupling wash and Fmoc-deprotection the peptidyl resin is ready for drug conjugation.

*Loading of amino acid Fmoc Lys (Dde)OH.* To resin with the provides described sequence (0.300 mg, 0.168 mmol loading) in a jacketed fritted peptide vessel was added a solution of protected amino acid Fmoc-Lys-(Dde)-OH (0.268 mg, 0.504 mmol) in NMP (3.5 ml), and after addition of DIPEA (0.165 ml, 1.01 mmol) the mixture was shaken for 1.5 h. After that, usual washings with NMP (5 times, 7 ml, 2 min each time) were applied to afford resin for ready for the next step.

*Loading of CLB and CAMP.* After post coupling wash and Fmoc-deprotection CLB (156 mg, 0.504 mmol), DIPEA (0.165 ml, 1.01 mmol) and coupling reagent PyBop (262 mg, 0.504 mmol) were pre-activated in NMP (3.5 ml each) for 2 min at rt in usual manner and added to the peptidyl resin and shaken for 2 h). Completion of the reaction was monitored by ninhydrin test (Kaiser test, yellow). DDE group was removed by treatment with 2% hydrazine in DMF (2 × 3min, 3.5 mL each) and subsequent usual washings with NMP (5 times, 7 ml, 2 min each time), obtaining deprotected peptidyl resin ready for the next step CAMP–CO_2_C_6_H_4_–*p*-(NO_2_) (0.258 mg, 0.504 mmol) were dissolved in DMF (3.5 ml) and DIPEA (0.165 ml, 1.01 mmol), and then the pre-activated compound was added to the resin for coupling and shaken for 2h at rt. Then the resin was washed with NMP (5 times, 7 ml, 2 min each time). After the usual work up washing with (3 × DCM, 5 ml each) the resin dried under the nitrogen and transferred to a vial for cleavage.

*General Procedure for cleavage of loaded peptidyl platforms from Cl-Trt (2-Chlorotritylchloride) resin.* A cold cleavage solution TFA (Trifluoroacetic acid)/triisopropylsilane/H_2_O 95:2.5:2.5, 5 ml) was added to the dried resin in the cleavage vessel. After shaking for 2 h, the solution was collected, and the resin washed with cold TFA (2 × 1 ml each). After combining the TFA solutions, the solvent was evaporated under an N_2_ stream and then precipitated by diethyl ether, purified by preparative HPLC on RP-18 (reverse phase-18). After purification, the collected fraction with the desired product was lyophilized to give PTR-58-CAMP-CLB. Analytical data: yield (87%), Purity (HPLC, 81%), LCMS m/z calcd for C_106_H_133_Cl_2_N_21_O_18_ S_2_ (Ms2H^+^) 2123.90, found (Ms/2) 1061.4. Labelling of the compound with the fluorescent dye have been done using the BODIPY-FL.

### 2.3 Methods

#### 2.3.1 Fluorescence spectroscopy

The fluorescence spectra recording procedure is described in [1].

#### 2.3.2 Optical microscopy

Images obtaining procedure is described in [1].

#### 2.3.3 Staining of the cells for detection of changes in morphology

The procedure of cells preparation is described in [1].

#### 2.3.4 High-performance liquid chromatography (HPLC)

All HPLC purifications were done via reverse phase on ECOM semi-preparative system with TOPAZ dual UV detection at 254 nm and 230 nm. Phenomenex Gemini^®^ 10 μm C18 110 Å, LC 250 × 21.2 mm column was utilized. The column was kept at room temperature. Peaks were detected at 220 nm and 280 nm. Analytical RP-HPLC was performed on an UltiMate 3000 system (Dionex) using a Vydac C18 column (250 × 4.6 mm) with silica (300 Å pore size) as a stationary phase. Linear gradient elution with eluent A (0.1% TFA in water) and eluent B (Acetonitrile) was used at a flow rate of 1 mL/min. Peaks were detected at 254 nm.

#### 2.3.5 Liquid chromatography mass spectrometry (LCMS)

Electron spray ionization mass spectra (ESI-MS) were obtained using an Autoflex III smart-beam (MALDI, Bruker), Q-TOF micro (Waters) or LCQ FleetTM ion trap mass spectrometer (Finnigan/Thermo). HPLC/LC-MS analyses were made using Agilent infinity 1260 connected to Agilent quadruple LC-MS 6120 series equipped with ZORBAX SB-C18, 2.1 × 50 mm, 1.8 μm HPLC column. In all cases, the eluent solvents were A (0.1% Formic acid in H_2_O) and B (100% CH_3_CN). The UV detection was at 254 nm. The column temperature was kept at 50 °C. The flow rate was 0.4 ml/min. The MS fragmentor was tuned on 30 V or 70 V in positive or negative mode.
