# Supplementary material for: Data on peptidyl platform-based anticancer drug synthesis and triton-x-based micellar clusters (MCs) self-assembly peculiarities for enhanced solubilization, encapsulation of hydrophobic compounds and their interaction with HeLa cells
Source: Data Brief. 2019 May 24;25:104052. doi: 10.1016/j.dib.2019.104052 (PMC6556622; doi:10.1016/j.dib.2019.104052)
Supplement: Multimedia component 1 [file mmc1.docx]

**Conflict of interest form for Data in Brief Journal**

All authors have participated in (a) conception and design, or analysis and interpretation of the data; (b) drafting the article or revising it critically for important intellectual content; and (c) approval of the final version.

🗹 The Article I have submitted to the journal for review is original, has been written by the stated authors and has not been published elsewhere.

🗹 The Images that I have submitted to the journal for review are original, was taken by the stated authors, and has not been published elsewhere.

🗹 This manuscript has not been submitted to, nor is under review at, another journal or other publishing venue.

All authors have read and approved this version of the article, and due care has been taken to ensure the integrity of the work. We attest that each author has made a significant scientific contribution to the study and has assisted with the drafting or revising of the manuscript, by the definition of an author. We confirm that neither the manuscript nor any part of it has been published or is under consideration for publication elsewhere. Any reference to or use of previously published material protected by copyright is explicitly acknowledged in the manuscript. Authors declare no conflict of interests.
