# Supplementary material for: Data on peptidyl platform-based anticancer drug synthesis and triton-x-based micellar clusters (MCs) self-assembly peculiarities for enhanced solubilization, encapsulation of hydrophobic compounds and their interaction with HeLa cells
Source: Data Brief. 2019 May 24;25:104052. doi: 10.1016/j.dib.2019.104052 (PMC6556622; doi:10.1016/j.dib.2019.104052)
Supplement: Supplementary file 2 — Multimedia component 2 [file mmc2.docx]

**SUPPORTING INFORMATION**

| Range of Triton X stock concentration causing micellar clusterization, mM | 0.2 – 4 (TX-100)  0.2 – >12 (TX-114) |
| --- | --- |
| Range of BPhen stock concentration causing micellar clusterization, mM | 5 – 20 |
| Range of metal ions stock concentration causing micellar clusterization, mM | 0.1 – 50 |
| Range of ambient electrolyte stock concentration causing micellar clusterization, mM | 400 – >1600 (TX-100)  100 – >1600 (TX-114) |
| Solvents, applicable for BPhen dissolution prior to addition to the surfactants | Methanol, Ethanol, 1-Propanol, Acetonitrile, Dimethylsulfoxide, Dimethylformamide |
| Efficient surfactant-to-chelator ratio causing micellar clusterization | 1.35 – 2.85 (TX-100)  0.35 – 2.85 (TX-114) |
| Efficient metal ion-to-surfactant ratio causing micellar clusterization | 9 to 1 – 1 to 9 |
| Availability of the additional ions/organic compounds adding to the formed MCs | Available |
| The time required to produce stable clusters | Within 1-2 hours at 19-25 °C |
| Metal ions used for micellar clusterization | Fe^2+^ (clusters color – red)  Ni^2+^, Cu^2+^, Zn^2+^, Mn^2+^  (clusters color – transparent) |
| Period of clusters stability | Up to 1 week at 18-23 °C at the moist atmosphere without drying |
| Availability of the clusters size variation and stabilization | Available |
| The range of the MCs size | ~1 – ~30 *µ*m (TX-100)  ~1 – ~700 *µ*m (TX-114) |
| MCs stability at elevated temperatures | Stable at 18-37 °C. At 37 °C stable at least 2 days |
| Scalable/non-scalable | Scalable, the volume of the MCs that could be obtained is from 10 *µ*l up to 200 *µ*l |
| Encapsulation abilities | Hydrophobic fluorescent compounds, anticancer drugs |

**Table S1.** Summary of the most useful parameters for Triton-X-based MCs synthesis and followed encapsulation of the peptide anticancer drugs and hydrophobic compounds.


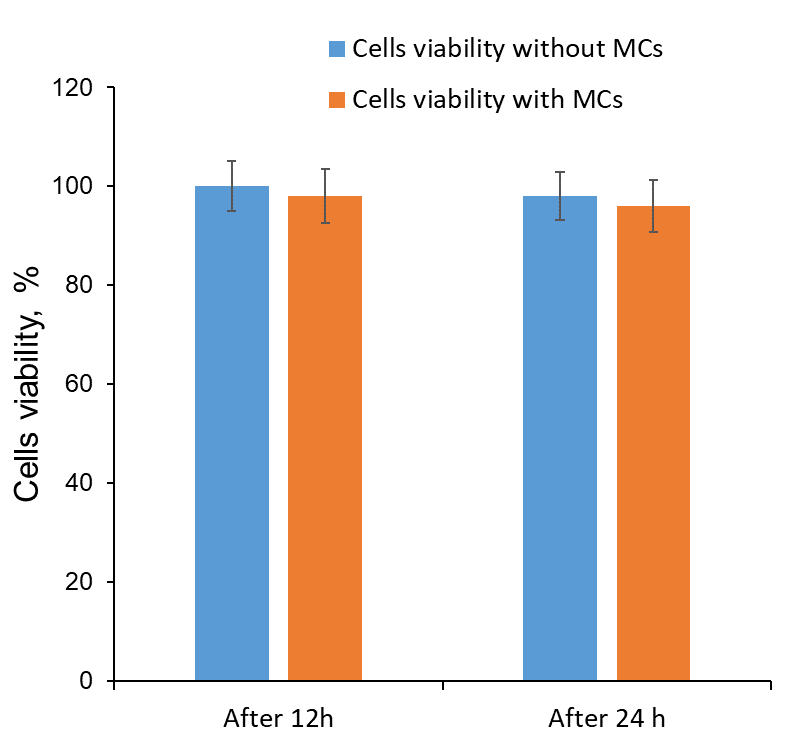


**Figure S1.** The HeLa cells viability with and without the presence of drug-free MCs. The cells viability/counting experiment was performed with BioRad TC-20 automated cell counter by pipetting of 10 *µ*l of the cells suspension into the outer opening of the chamber of the counting slide.
